# Machine Learning Classification Combining Multiple Features of A Hyper-Network of fMRI Data in Alzheimer's Disease

**DOI:** 10.3389/fnins.2017.00615

**Published:** 2017-11-21

**Authors:** Hao Guo, Fan Zhang, Junjie Chen, Yong Xu, Jie Xiang

**Affiliations:** ^1^Department of Software Engineering, College of Computer Science and Technology, Taiyuan University of Technology, Taiyuan, China; ^2^Department of Psychiatry, First Hospital of Shanxi Medical University, Taiyuan, China

**Keywords:** fMRI, hyper-network, multi-feature, discriminative subgraph, Alzheimer's disease

## Abstract

Exploring functional interactions among various brain regions is helpful for understanding the pathological underpinnings of neurological disorders. Brain networks provide an important representation of those functional interactions, and thus are widely applied in the diagnosis and classification of neurodegenerative diseases. Many mental disorders involve a sharp decline in cognitive ability as a major symptom, which can be caused by abnormal connectivity patterns among several brain regions. However, conventional functional connectivity networks are usually constructed based on pairwise correlations among different brain regions. This approach ignores higher-order relationships, and cannot effectively characterize the high-order interactions of many brain regions working together. Recent neuroscience research suggests that higher-order relationships between brain regions are important for brain network analysis. Hyper-networks have been proposed that can effectively represent the interactions among brain regions. However, this method extracts the local properties of brain regions as features, but ignores the global topology information, which affects the evaluation of network topology and reduces the performance of the classifier. This problem can be compensated by a subgraph feature-based method, but it is not sensitive to change in a single brain region. Considering that both of these feature extraction methods result in the loss of information, we propose a novel machine learning classification method that combines multiple features of a hyper-network based on functional magnetic resonance imaging in Alzheimer's disease. The method combines the brain region features and subgraph features, and then uses a multi-kernel SVM for classification. This retains not only the global topological information, but also the sensitivity to change in a single brain region. To certify the proposed method, 28 normal control subjects and 38 Alzheimer's disease patients were selected to participate in an experiment. The proposed method achieved satisfactory classification accuracy, with an average of 91.60%. The abnormal brain regions included the bilateral precuneus, right parahippocampal gyrus\hippocampus, right posterior cingulate gyrus, and other regions that are known to be important in Alzheimer's disease. Machine learning classification combining multiple features of a hyper-network of functional magnetic resonance imaging data in Alzheimer's disease obtains better classification performance.

## Introduction

Modern imaging techniques provide effective approaches for exploring the functional interactions among brain regions, increasing our understanding of the pathological basis of mental illnesses. Brain functional network approaches provide a simplified representation of brain interaction patterns, and have been successfully used to classify neurological disorders (Stam et al., 2009; Pievani et al., 2011; Wang et al., 2013). The application of brain functional networks to neurocognitive theory has attracted much attention and recognition from researchers (Richardson, 2010), and they are widely used in the study of brain diseases, including schizophrenia (Bassett et al., 2008; Liu et al., 2008; Lynall et al., 2010), depression (Liu F. et al., 2015), mild cognitive impairment (Liang et al., 2013), attention deficit hyperactivity disorder (Wang et al., 2009), and Alzheimer's disease (AD) (He et al., 2008; Supekar et al., 2008).

Because network structures are composed of nodes and edges, functional brain network analysis provides an important tool for systematically detecting abnormalities in several brain regions. Differences in network topology between normal controls and brain disease patients can provide useful biomarkers for diagnosis, and for understanding the pathological underpinnings of brain diseases. Thus, modeling of functional networks can play an essential role in accurate diagnosis. Many previous studies have reported that higher cognition arises from interactions among many different brain regions, rather than activities in isolated brain regions. A major symptom in many mental disorders is a sharp decline in cognitive ability, which can be related to abnormal connectivity patterns (Delbeuck et al., 2003; Horwitz, 2003) involving interactions among multiple brain regions.

So far, many functional connectivity modeling methods have been proposed, including correlation-based methods (Bullmore and Sporns, 2009), graphical models (Bullmore et al., 2000), partial correlation-based methods (Rosa et al., 2015), and sparse representation methods (Smith et al., 2011; Wee et al., 2014). However, there are some flaws in the conventional methods of constructing functional networks. Most of them use correlation-based methods, which are relatively sensitive for detecting network connections (Smith et al., 2011). Nevertheless, because most network modeling methods are based on correlations, they are only able to reflect relationships between paired brain regions, which does not fully characterize the multi-level information among multiple brain regions, and ignores the higher-order relationships that are important for disease diagnosis. Moreover, network models based on correlational methods may contain false connections, because of the arbitrary selection of thresholds (Biao et al., 2014; Jie et al., 2016). Other methods of studying brain connectivity have been proposed, including graphical models such as structural equation models (Mcintosh et al., 1994; Bullmore et al., 2000) and dynamic causal models (Marreiros et al., 2010). However, most of these methods are confirmative rather than exploratory, which makes them inadequate for studying brain connectivity in AD and mild cognitive impairment (MCI) because they often require a prior knowledge—such as which brain regions should be involved and how they are connected—that is usually unavailable (Huang et al., 2010). Partial correlation estimation can be implemented using the maximum likelihood estimation (MLE) of the inverse covariance matrix. However, using this method, the required sample size to obtain sufficient data for reliable estimation is much larger than the number of modeled brain regions (Jie et al., 2016).

Conventional methods for constructing functional networks typically model the relationships between pairwise brain regions. However, recent studies have reported the importance of interactions among multiple brain regions, in addition to the relationships between pairwise brain regions. In one study, Yu et al. (2011) demonstrated that higher-order interactions are inherent properties of cortical dynamics. Santos et al. (2010) reported that the recorded activity of units in pairwise interactions was not best described by neuronal activity patterns. To address this limitation, they constructed a hierarchical model of network interactions, using units of interactions at two spatial levels. The results suggested that hierarchical models can capture network interactions more accurately than pairwise models. Montani et al. (2009) modeled the impact of high-order interactions on the amount of somatosensory information transmitted by the rate of synchronous discharge. Taken together, these results suggest that higher-order interactions play an important role in the dynamics of neural networks. Moreover, some studies have also suggested that functional interactions among single brain regions can interact with several other brain regions (Huang et al., 2010). Therefore, correlational analysis reflecting pairwise information may not be able to characterize the higher-order interactions of many brain regions working together. However, this information may be crucial for understanding the pathological mechanisms underlying mental illness.

In view of the shortcomings of the conventional functional connectivity network models, many new methods of construction have been developed. Hyper-networks are one example. A properly constructed hyper-network can overcome the above disadvantages of conventional methods. Hyper-networks based on hyper-graph theory can represent the interactions among multiple brain regions (Biao et al., 2014; Jie et al., 2016). Recently, Jie et al. (Biao et al., 2014) constructed a hyper-network for an MCI dataset, extracted the local brain region properties as features, and then selected the most discriminative features for classification. Jie et al. (2016) similarly constructed a hyper-network for an attention deficit hyperactivity disorder (ADHD) dataset and extracted the brain region properties as features. They compared the hyper-network results with those of the conventional functional connectivity network methods and verified the robustness of various technologies.

However, the above classification methods extracted the local brain region properties as features, so that feature selection and classification could be implemented. Brain region features, including global properties (clustering coefficient Dj and Sh, 1998, path length Saramäki et al., 2006, etc.) and local properties (degree, betweenness centrality Barthélemy, 2004, etc.), have been widely used in previous studies for the classification of diseases in connectivity networks. However, such extracted features may lose some useful information, especially global topological information (Zhou et al., 2014). Subgraph features that are extracted from the graph-structure have been widely applied in the diagnosis of brain diseases (Montani et al., 2009; Huang et al., 2010) and can effectively compensate for the defects of conventional feature-extraction methods. However, subgraph feature-based methods have the drawback of being insensitive to change in a single brain region (Zhou et al., 2014). Therefore, both types of methods can lead to the loss of sample information (Zhou et al., 2014). In addition, the brain network itself is a complex network structure and its biological features cannot be captured from the perspective of a single feature.

To solve the problems of conventional methods, we developed a novel method that uses machine learning classification to combine multiple features of hyper-network functional magnetic resonance imaging (fMRI) data in AD. Specifically, based on the resting-state fMRI time sequence, we constructed a hyper-network with a sparse representation method. In the current study, to address the limitations of conventional network modeling, we combined different types of features, including brain region features and subgraph features. Three types of clustering coefficients were extracted as features and a non-parametric test was applied for feature selection. The subgraph feature-based method extracted hyper-edges as features and selected them using the frequently scoring feature selection (FSFS) method. Finally, two types of kernels based on multi-kernel support vector machine (SVM) classification were combined. The study constructed hyper-networks for 38 AD patients and 28 normal subjects and verified them. The results showed that the proposed machine learning classification method combining multiple features of a hyper-network of fMRI data in AD achieved satisfactory classification performance.

The main work of this study was as follows. First, the hyper-network construction method was applied to construct a network model based on an AD dataset to analyze the interactions among multiple brain regions. Second, different from previous studies, this study extracted two types of hyper-network features—brain region features and subgraph features—to ensure the integrity of the network topology information and preserve the sensitivity to change in a single brain region. Third, a multi-kernel SVM was proposed for the hyper-network, which combines two types of network features to achieve better classification performance.

## Materials and methods

### Method framework

**Figure 1:**
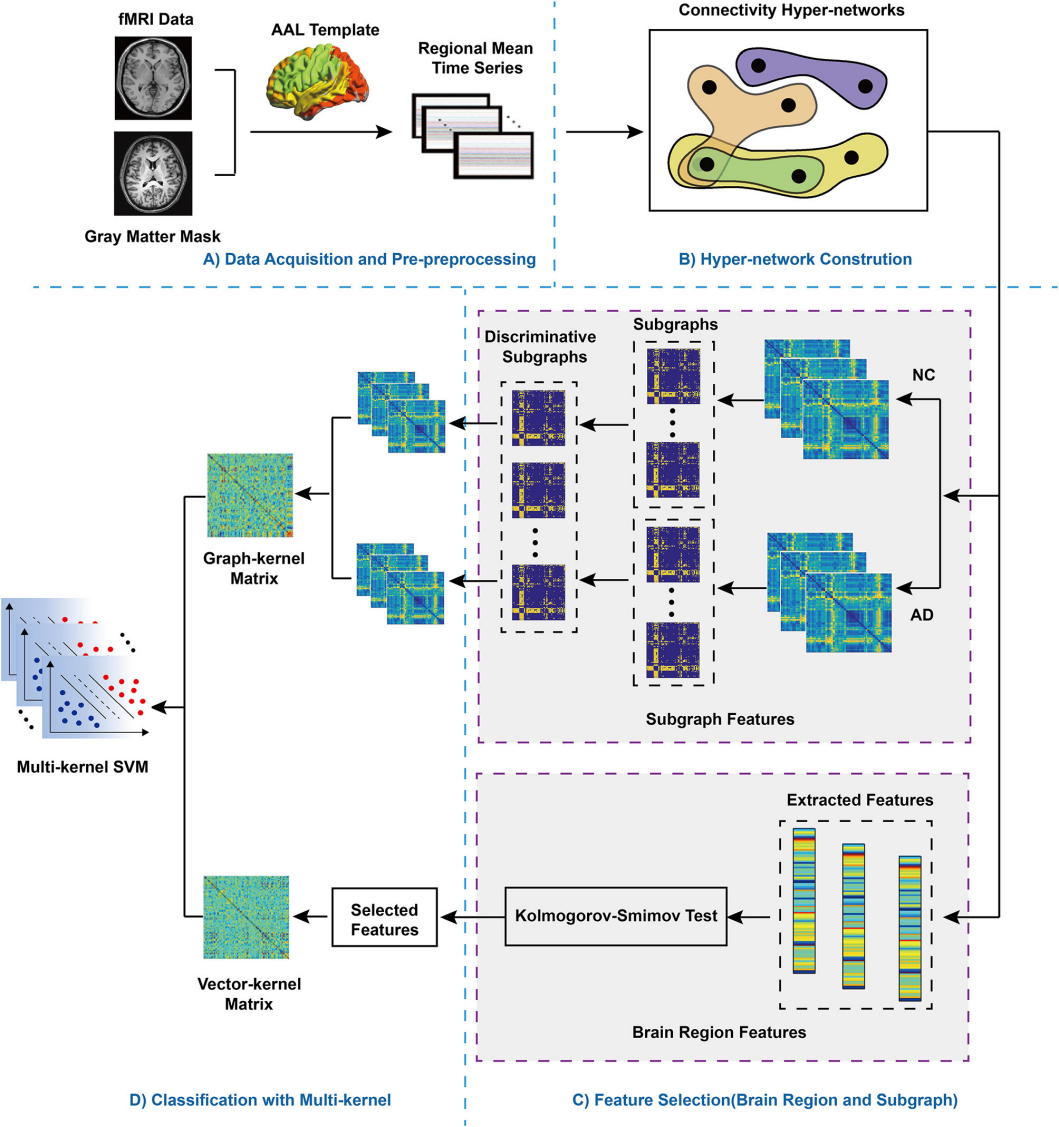
Flowchart of the proposed method. **(A)** Data Pre-processing. AAL: automatic anatomic labeling. fMRI: functional magnetic resonance imaging. After fMRI data pre-processing, according selected AAL template, the whole brain was divided into 90 regions. Then, the mean regional time series were extracted to divided brain regions, **(B)** Hyper-network Construction. **(C)** Feature Selection (brain region and subgraph). For subgraph features, NC, normal control; AD, Alzheimer's disease, hyperedges were extracted respectively from the hyper-networks contructed the NC group and the AD group, which were regarded as subgraph features, so two groups of subgraph features were obtained. In addition, for brain region features, the values of three different clustering coefficient respectively were computed, then Kolmogorov-Smimov test was adopted to feature selection, which obtained discriminative features. **(D)** Classification with multi-kernel SVM. Two different types of kernel matrix were combined, adopting multi-kernel SVM for classification.

A flowchart of the proposed framework for machine learning classification combining multiple features of a hyper-network of fMRI data in AD is presented in Figure 1. Specifically, the framework consists of several major steps.

1. Data acquisition and pre-processing.
2. Construction of the hyper-network: for each subject, we constructed a hyper-network using a sparse linear regression model that estimated a region using a linear combination of the times series of other regions, and optimized the objective function by sparse learning.
3. Feature extraction and selection: non-parametric tests were performed to select the brain region features and the FSFS algorithm was used to select discriminative subgraphs; then, the corresponding kernel matrix was computed.
4. Multi-kernel SVM: multi-kernel SVM was used for classification of the kernel matrixes with brain region features and subgraph features combined.

### Data acquisition and pre-processing

This study was carried out in accordance with the recommendations of the medical ethics committee of Shanxi province (reference number: 2012013). All subjects gave their written informed consent in accordance with the Declaration of Helsinki. Twenty-eight healthy right-handed participants and thirty-eight major depression disorder participants underwent resting-state fMRI in a 3T MR scanner (Siemens Trio 3-Tesla scanner, Siemens, Erlangen, Germany). The subjects' demographic information and clinical characteristics are summarized in Table 1. Data collection was completed at the First Hospital of Shanxi Medical University. All scans were performed by radiologists who were familiar with MRI. All patients underwent a complete physical and neurological examination, standard laboratory tests, and an extensive neuropsychological assessment battery.

**Table 1:** Demographics and clinical characteristics of the subjects.

	**NC**	**AD**	***P*-value**
Age(years)	(68–76) 72.6 ± 3.42	(66–76) 71.4 ± 4.68	0.44^a^
Sex (male/female)	13/15	15/23	0.57^b^
Handedness (R/L)	28/0	38/0	–
MMSE	(23–30) 26.1 ± 3.2	(20–25) 22.8 ± 2.1	<0.0001^a^

Values are mean + standard deviation; AD, Alzheimer's disease; NC, normal controls; MMSE, Mini-Mental State Examination; ^*^All tests are two-tailed;

a Two-sample t-test;

b *Pearson Chi-square test*.

During the scan, participants were asked to relax with their eyes closed but not to fall asleep. The scanning parameters were set as follows: axial slices = 33, repetition time (TR) = 2,000 ms, echo time (TE) = 30 ms, thickness/skip = 4/0 mm, field of view (FOV) = 192 × 192 mm, matrix = 64 × 64 mm, flip angle = 90°, volumes = 248. The first 10 volumes of each time series were discarded to allow for magnetization stabilization. See Supplemental Text S1 for details of the scanning parameters.

Data preprocessing was performed with SPM8 (Statistical Parametric Mapping, SPM) (Friston, 2007). First, slice-timing correction and head-movement correction were carried out. Two samples exhibiting more than 3.0 mm of translation and 3.0° of rotation were discarded, which were not included in the final 28 samples. The corrected images were optimized with a 12-dimensional affine transformation and normalized to 3 × 3 × 3 mm voxels in the Montreal Neurological Institute (MNI) standard space. Finally, linear detrending and band-pass filtering (0.01–0.10 Hz) were performed to reduce the effects of low-frequency drift and high-frequency physiological noise.

### Construction of the hyper-network

Most previous studies have used the simple-graph to construct network models, which only characterize information between pairwise brain regions. In the current study, we constructed a hyper-network connectivity model based on hyper-graph theory, which can reflect higher-order interactions among multiple brain regions. A hyper-graph is an expansion based on a simple-graph, and the approach has been widely used in numerous fields. The hyper-graph is summarized as follows.

#### Hyper-graphs

To date, hyper-graph theory has been successfully used for many applications, including image classification (Yu et al., 2012) and protein function prediction (Gallagher and Goldberg, 2013). In the field of neuroimaging, graph theory is commonly used to analyze brain connectivity (Kaiser, 2011; Sporns, 2012; Fornito et al., 2013). In the traditional graph theory approach, every edge merely links two nodes with a particular relationship, meaning that it only reflects the interactions between two nodes. In addition to paired relationships, such as functional interactions among multiple brain regions, many scenarios involve higher-order relationships, which simple graphs cannot describe. To address this limitation, some researchers have proposed the use of hyper-graphs, which are able to reflect the higher-order relationships among multiple nodes. Generally, a hyper-graph can be represented by an extension of a conventional simple graph in which one hyper-edge links two or more nodes (Schölkopf et al., 2007).

A hyper-graph is represented by *G* = (*V, E*), where *V* denotes a set of nodes and *E* represents a set of hyper-edges. We can then use a |*V*| × |*E*| incidence matrix *H* to denote *G*, where *H* is represented by the following elements:

(1) $$\begin{array}{l} H\left(\nu ,e\right)=\{\begin{array}{ll} 1, & if\;v\in e\\ 0, & if\;v\notin e \end{array} \end{array}$$

where *v* ∈ *V* indicates a node of *G*, and *e* ∈ *E* indicates a hyper-edge of *G*.

Based on *H*, the node degree of each node *v* ∈ *V* can be represented as:

(2) $$\begin{array}{l} d\left(v\right)\;=\;\sum_{e\in E}H\left(v,e\right) \end{array}$$

The edge degree of hyper-edge *e* ∈ *E* can be represented as:

(3) $$\begin{array}{l} \delta \left(e\right)\;=\;\sum_{v\in V}H\left(v,e\right) \end{array}$$

Let *D*_*v*_ and *D*_*e*_ represent the diagonal matrices of node degrees *d*(*v*) and hyper-edge degrees δ(*e*):

(4) $$\begin{array}{l} A=HH^{T}-D_{v} \end{array}$$

where *H*^*T*^ is the transpose of *H. A*(*i, j*) represents the number of hyper-edges that contain nodes c*v*_*i*_ and *v*_*j*_.

**Figure 2:**
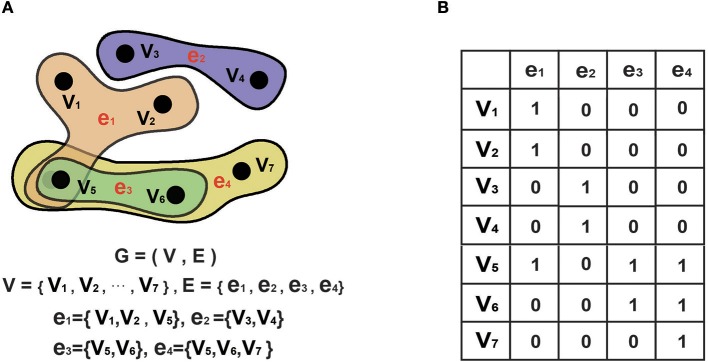
Hyper-graph. **(A)**. A hyper-graph. G denotes the graph, V denotes the set of nodes. E denotes the set of edges. In a hyper-graph, multiple nodes can be connected toghter by each hyper-edge. **(B)** The corresponding incidence matrix of the hyper-graph in A. 0 represents no connection between nodes of the corresponding row and column, and 1 represents connection between them.

Notably, the traditional graph is a specific hyper-graph where one hyper-edge includes only two nodes. An example of a hyper-graph is shown in Figure 2: Figure 2A displays a conventional graph; Figure 2B shows a hyper-graph; and Figure 2C is an incidence matrix for the hyper-graph in Figure 2B, where 0 indicates that there is no connection between the nodes in the corresponding row and column and 1 indicates that there is such a connection.

#### Construction of hyper-networks

Based on the sparse linear regression model, hyper-networks were constructed from rs-fMRI time series (Mcintosh et al., 1994). Specifically, $X={\left[x_{1},\cdots \;,x_{m},\cdots \;,x_{M}\right]}^{T}\in R^{M\times d}$ is used to denote subjects with a total of *M* regions of interest (ROIs), where *x*_*m*_ denotes the regional mean time series of the designated *m*-th ROI, and *d* is the length of the time series. A response vector *x*_*m*_ is then denoted by the regional mean time series of each ROI, which can be represented by adopting a linear combination of the time series of other *M* − 1 ROIs, as follows:

(5) $$\begin{array}{l} x_{m}\;=\;A_{m}\alpha_{m}+\tau_{m},\;m\;=\;1,2,\cdots \;,M \end{array}$$

where *A*_*m*_ = [*x*_1_, ⋯ , *x*_*m*−1_, 0, *x*_*m*+1_, ⋯ , *x*_*M*_] denotes a data matrix that contains all time series except the *m*-th ROI, in which the regional mean time series was set a vector of all zeros), α_*m*_ denotes the weight vector to indicate the degree of the effect of other ROIs on the *m*-th ROI, and${\;\tau }_{m}\in R^{d}$ represents a noise term. It should be noted that the element in α_*m*_ indicates that its corresponding ROI is meaningless for accurately evaluating the time series of the *m*-th ROI.

For solving sparse linear regression models, this optimization target function is as follows:

(6) ‖xm−Amαm‖αmmin2+λ‖αm‖0

This is a well-known non-deterministic polynomial (NP) problem, owing to the *l*_0_-norm term. Meanwhile, this method is usually approximately equal to solving a standard *l*_1_-norm regularized optimization problem through a target function (Wright et al., 2009), as follows:

(7) ‖xm−Amαm‖αmmin2+λ‖αm‖1

where λ > 0 denotes a regularization parameter to control the levels of sparsity of the model. Different λ values correspond to different solutions of the degree of sparsity, and a larger λ value represents a sparser model, which indicates that there are more zero elements in α_*m*_. Most existing sparse learning algorithms can be implemented to solve the *l*_1_-norm, such as least angle regression (Statistics, 1998). Adopting the sparse linear regression model, we can obtain one brain region's interactions with other brain regions, while setting the irrelevant or false connections to zero. This means that, in the weight vector α_*m*_, the brain regions corresponding to zero elements are regarded as irrelevant for estimating one region's time series. Thus, this approach provides a method for modeling interactions among one brain region and other brain regions by eliminating irrelevant connections.

In this study, for each subject, we constructed a hyper-network by adopting a sparse linear regression model, where a node is represented by one brain region, and a hyper-edge *e*_*m*_ contains the *m*-th ROI and other ROIs with corresponding non-zero elements in the weight vector α_*m*_, which is computed by Equation (7). As a regularization parameter, λ controls the amount of non-zero solutions of the sparistiy vector α_*m*_. In the extreme situation, when α_*m*_ obtains all the zero solutions, λ could get the maximum value which is always denoted as λ_max_. On the contrary, when α_*m*_ obtains all the non-zero solutions, λ could get the minimum value which is a positive number close to 0, denoted as λ_min_. Thus the value of λ should be set ranging from λ_min_ to λ_max_ (Lee et al., 2011; Li et al., 2012). One of the limitations of the above setting method iss that different experimental data could achieve different λ_min_ and λ_max_. It make the parameter λ hard to be compared among different methods. Previous research standardized the range of λ from 0 to 1 based on λ_min_ and λ_max_ that made λ comparable (Liu et al., 2013). In the current study, we follow the latter setting method. When λ is more close to 0, there are more non-zero solutions in the α_*m*_ which suggests that there are more nodes in the given hyper-edge. Otherwise, the opposite. Besides, to characterize the multi-level relationships within multiple brain regions, an array of hyper-edges can be obtained by setting different values of λ within a required range for each node. Thus, multi-level relationships indicate that different values of λ mean different levels of information within multiple brain regions. That is, a target function shown in Equation (7), which reflects a larger value of λ, can obtain a sparser solution and thus the hyper-edge only includes some ROIs (i.e., nodes). We conducted tests to set different values of λ, ranging from 0.1 to 0.9 with a step of 0.1. Notably, in Equation (7), the values of weight vector α_*m*_ are the same for brain regions within the same time series. Therefore, they will simultaneously be contained or excluded in the hyper-edge corresponding with them. In the current study, we were able to obtain the optimal solution in Equation (7) by using the SLEP package (Liu et al., 2013).

### Feature extraction and selection

After constructing hyper-network, we investigated two types of network features: brain region features and subgraph features. The two types of features were then selected: the Kolmogorov-Smirnov non-parametric test was used for selecting quantifiable brain region features and the FSFS algorithm was used for selecting discriminative subgraph features.

#### Brain region features and feature selection

To quantify the local brain region properties of the hyper-network, three local clustering coefficients—HCC_1_, HCC_2_, and HCC_3_ (Gallagher and Goldberg, 2013)—were adopted, as they describe the local aggregation of the hyper-network from different angles. Table 2 shows the definitions and calculation formulas of these properties.

**Table 2:** The definitions and formulas of hyper-network properties.

**Properties**	**Definitions**	**Formulas**
HCC_1_	The number of adjacent nodes that have connections not facilitated by node ν	$HCC_{1}\left(\nu \right)=\frac{2\underset{u,t\in N\left(\nu \right)}{\sum }I\left(u,t,\neg \nu \right)}{|N\left(\nu \right)|\left(\left|N\left(\nu \right)-1\right|\right)}$
HCC_2_	The number of adjacent nodes that have connections facilitated by node ν	$HCC_{2}(\nu )=\frac{2\sum_{u,t\in N(\nu )}I^{\prime }(u,t,\nu )}{\left|N(\nu )|(|N(\nu )-1|\right)}$
HCC_3_	The amount of overlap among adjacent hyper-edges of node ν	$HCC_{3}\left(\nu \right)=\frac{2\underset{e\in S\left(\nu \right)}{\sum }\left(|e|-1\right)-|N\left(\nu \right)|}{|N\left(\nu \right)|\left(\left|S\left(\nu \right)-1\right|\right)}$

A multiple linear regression method was adopted to eliminate the influence of confounding factors of age, gender, and educational status for every network property (independent variable: the area under the curve (AUC) value of every network property; dependent variables: age, gender, and educational status). These results indicated that the relationship between network properties and confounding factors was not significantly relevant (see Supplemental Table T1 for the detailed results).

To select the discriminative features, the Kolmogorov-Smirnov non-parametric test (Fasano and Franceschini, 1987) was used to select the quantifiable local brain region properties, corrected by the false-discovery rate (FDR) (Benjamini and Hochberg, 1995) (q = 0.05) method. The brain region features with *P* < 0.05 (FDR correction) were selected as discriminative features. Finally, we obtained a kernel matrix according to the above selected features.

#### Subgraph features and feature selection

The hyper-edges are regarded as the subgraph features of the hyper-network. The number of subgraphs is very large, but only a few features are truly discriminative. Accordingly, in this study, we selected the most discriminative subgraphs as features to be used in the classification in the next step. Detailed information on the discriminative subgraphs can be found in the Supplemental Text S2.

Discriminative subgraphs can be regarded as features for classification (Kong et al., 2013). However, because the subgraph features extracted from the normal control (NC) group and the AD group may not have discriminative ability, adopting only the extracted subgraph features would degrade the classification performance. To solve this problem, first, we used the discriminative score of the subgraph pattern (Santos et al., 2010) to complete the initial feature selection, also referred to as FSFS. This method calculates the discriminant scores of these subgraphs mined from the NC and AD groups and sorts them. The most discriminating scores *t*_1_, *t*_2_ are selected as discriminant subgraphs.

Formally, we introduce the following notation:

*D*:D = {*D*_*n*_, *D*_*p*_}, where *D*_*n*_ represents the negative samples, and *D*_*p*_ represents the positive samples.

*G*:*G* = {*G*_*n*_, *G*_*p*_}, where *G*_*P*_ = {*g*_*p*1_, *g*_*p*2_, ⋯ , *g*_*pm*_} denotes a set of all subgraph features in positive samples, and *G*_*n*_ = {*g*_*n*1_, *g*_*n*2_, ⋯ , *g*_*nk*_} denotes a set of all subgraph features in negative samples.

T^*^: The optimal set of subgraph features, $T^{*}=T_{1}^{*}\cup T_{2\;}^{*}$ and $T_{1}^{*}\subseteq G_{p}$,$T_{2}^{*}\;\subseteq G_{n}$; hence, T^*^ ⊆ *G*.

*J*(*T*): The criteria to evaluate the effectivity of subgraph feature subset *T*.

*S*(*g*_*s*_): The discriminative score of a subgraph pattern *g*_*s*_is defined as follows:

(8) $$\begin{array}{l} S\left(g_{s}\right)=|f_{q}\left(g_{s}|D_{p}\right)-f_{q}\left(g_{s}|D_{n}\right)| \end{array}$$

The discriminative score of subgraph *g*_*s*_means its frequency difference between positive samples and negative samples, that is, the bigger the *S*(*g*_*s*_), the bigger the difference of these subgraphs between the AD and NC groups. *S*(*g*_*s*_) = 0 denotes that this subgraph pattern *g*_*s*_ was not present in any graphs in the NC group, but was present in all graphs in the MDD group, or vice versa.

In this study, we obtained the optimal set of subgraph features according to Equation (9):

(9) T∗=T1⊆Gp,T2⊆GnJargmax(T)     s.t  |T1|≤t1,|T2|≤t2

where |·| denotes the size of the feature set, and *t*_1_,*t*_2_ are the maximum number of features selected from the NC and MDD groups, respectively. We can then obtain the following equation:

(10) $$\begin{array}{l} J\left(T\right)=\sum_{i\leq t_{1}}S\left(g_{pi}\right)+\sum_{j\leq t_{2}}S\left(g_{nj}\right) \end{array}$$

We can compute the discriminative score of each subgroup using Equation (8). Suppose the scores of all subgraphs are denoted as

(11) $$\begin{array}{l} S\left(g_{p}^{1}\right)\geq S\left(g_{p}^{2}\right)\cdots \geq S\left(g_{p}^{m}\right),S\left(g_{n}^{1}\right)\geq S\left(g_{n}^{2}\right)\cdots \geq S\left(g_{n}^{k}\right) \end{array}$$

Based on Equation (11), we can obtain the optimal set of subgraph features, as

(12) $$\begin{array}{l} {\;T}^{*}=\left\{g_{p}^{i},g_{n}^{j}|i\leq t_{1},j\leq t_{2}\right\} \end{array}$$

We obtained discriminative subgraphs based on the selected subgraph features by adopting the FSFS method. Due to the excessive number of discriminant subgraphs obtained by the FSFS method, we conducted a further selection using the threshold of discriminative score *K*.

### Construction of classification model

Because we used a combination of local brain region features and subgraph features as classification features, we adopted the multi-kernel SVM classifier based on the vector kernel and the graph kernel. For the vector kernel, we used the function-based RBF kernel, which is a widely used classification method (Cortes and Vapnik, 1995; Chen X. et al., 2011). The graph kernel is a common method for subgraph tests of isomorphism. It bridges the gap between graph-structured data and a large spectrum of machine learning algorithms called kernel methods (Borgwardt et al., 2005), which include algorithms such as support vector machines, kernel regression, or kernel principal component analysis (Hofmann et al., 2007). The graph kernel is outlined below.

#### Graph Kernel

Kernels are widely considered to be suitable indicators for evaluating the topological similarity of pairwise subjects. Kernels can map the data from an original space onto a higher dimensional feature space, generally causing the data to be more linearly separable. The corresponding kernel between subject *x* and *y* can be represented as follows:

(13) $$\begin{array}{l} k\left(x,y\right)=\langle \varphi \left(x\right),\varphi \left(y\right)\rangle \end{array}$$

where φ denotes a mapping function that can map data from the input space to the feature space. Many complex data types can be implemented through the kernel. The corresponding kernel of the graph is referred to as the graph kernel (Vishwanathan et al., 2008), which evaluates the topological similarity between paired graphs. Various methods have been proposed for constructing graph kernels, including walk-based (Gärtner et al., 2003), path-based (Alvarez et al., 2011), and subtree-based kernels (Shervashidze et al., 2011). Graph kernels have been successfully adopted for image classification (Harchaoui and Bach, 2007) and protein function prediction (Borgwardt et al., 2005). In the current study, we used the Weisfeiler-Lehman subtree kernel to measure the topological similarity between pairwise graphs. This method is implemented through the Weisfeiler-Lehman test of isomorphism (Shervashidze et al., 2011), which is described in detail in Supplemental Text S3.

#### Multi-Kernel SVM

Recent studies have shown that a multi-kernel SVM can more effectively integrate features from different modalities than a single kernel SVM (Vishwanathan et al., 2008). The combination of multiple kernels can improve classification performance, and can also increase the interpretability of the results (Lanckriet et al., 2002). In general, given two subjects *x* and *x*′, multiple kernels can be integrated by a linear combination method, as follows:

(14) $$\begin{array}{l} k\left(x,x^{\prime }\right)=\sum_{i=1}^{M}\alpha_{i}k_{i}\left(x,x^{\prime }\right)\;s.t\sum_{i=1}^{M}\alpha_{i}=1 \end{array}$$

where $k_{i}\left(x,x^{\prime }\right)$ denotes a basic kernel between *x* and *x*′, α_*i*_ denotes a weighting parameter (α_*i*_ > 0), and *M* denotes the number of combined kernel matrices.

In the current study, two types of kernel, based on a vector kernel and a graph kernel, were combined to construct the multi-kernel SVM classification model. However, when using two types of kernel for the classification, it was necessary to first implement one step separately to achieve normalization by computing Equation (15), then combining them.

(15) $$\begin{array}{l} {\;k}^{*}\left(x,x^{\prime }\right)=k\left(x,x^{\prime }\right)/\sqrt{k\left(x,x\right)k\left(x^{\prime },x^{\prime }\right)} \end{array}$$

Notably, in most studies of the multi-kernel method, the optimal weighting parameter *a*_*i*_ was simultaneously obtained with some other parameters. However, we adopted a grid search method to obtain *a*_*i*_. When *a*_*i*_ was determined, the multi-kernel SVM can be achieved by embedding the multi-kernel method into the conventional single-kernel SVM classifier.

In the current study, the multi-kernel SVM was used to implement the classification. We adopted the multi-kernel SVM method to effectively integrate multiple features, which fully described the overall interactive information of the brain network. Specifically, the vector kernel characterizes interactions among multiple brain regions using three different local cluster coefficients. Moreover, the graph kernel characterizes information about topological structure within the connectivity network.

#### Cross-validation

In the current experiment, we adopted 10-fold cross-validation (Chang and Lin, 2011) to evaluate the performance of our proposed classification method. Specifically, the subject dataset was randomly divided into 10 parts, one of which was left as the testing set, while the remaining nine were regarded as training sets. In this study, 10-fold cross validation was performed 100 times to obtain more accurate results. Finally, we computed the arithmetic mean of the 100 repetitions as the final result.

## Results

Two types of features were extracted and selected from the constructed networks, including brain region features and subgraph features. Brain region features computed and selected for the HCC_1_, HCC_2_ and HCC_3_. Subgraph features were selected by FSFS algorithm.

### Brain region features

After constructing the hyper-network, three local brain region properties, HCC_1_, HCC_2_, HCC_3_, were extracted and selected. Specially, HCC_1_ calculates the amount of adjacent nodes that have connections not facilitated by node *v*. HCC_2_ computes the amount of adjacent nodes that have connections facilitated by node *v*. HCC_3_ computes the number of overlap among adjacent hyper-edges of node *v*. The local brain region features and abnormal brain regions were then analyzed. Table 3 lists the abnormal brain regions and the significance of the brain region features. We used HCC_1_, HCC_2_, and HCC_3_, three local clustering coefficients, to indicate a significant difference (*p* < 0.05, FDR correction) in abnormal brain regions. Table 3 shows a total of 13 abnormal brain regions: the right middle frontal gyrus (MFG), left inferior temporal gyrus (ITG), right posterior cingulate gyrus (PCG), left supplementary motor area (SMA), right parahippocampal gyrus (PHG), right ITG, right precuneus (PCUN), left fusiform gyrus (FFG), left supramarginal gyrus (SMG), right hippocampus (HIP), right putamen (PUT), left thalamus (THA), and left middle temporal gyrus (MTG).

**Table 3:** The abnormal brain regions and significance of brain region feature.

**No**	**ROI**	**Name**	***P*****-values**		
			**HCC_1_**	**HCC_2_**	**HCC_3_**
1	Right	Middle frontal gyrus	**0.0199**	0.1578	0.1690
2	Left	Inferior temporal gyrus	0.1181	0.1181	**0.0077**
3	Right	Posterior cingulate gyrus	0.3033	0.0941	**0.0451**
4	Left	Supplementary motor area	0.2075	0.0557	**0.0077**
5	Right	Parahippocampal gyrus	0.6384	**0.0157**	**0.0059**
6	Right	Inferior temporal gyrus	**0.0173**	0.2601	0.1096
7	Right	Precuneus	0.0606	**0.0396**	**0.0451**
8	Left	Fusiform gyrus	0.7929	**0.0478**	0.6121
9	Left	Supramarginal gyrus	**0.0481**	0.2145	0.1181
10	Right	Hippocampus	0.3930	**0.0049**	0.0905
11	Right	Lenticular nucleus, putamen	0.2145	0.1226	**0.0478**
12	Left	Thalamus	0.2943	**0.0370**	0.2521
13	Left	Middle temporal gyrus	0.8049	**0.0379**	0.1813

*ROI denotes the reigon of interest. Bold indicates significance p < 0.05*.

### Subgraph features

**Figure 3:**
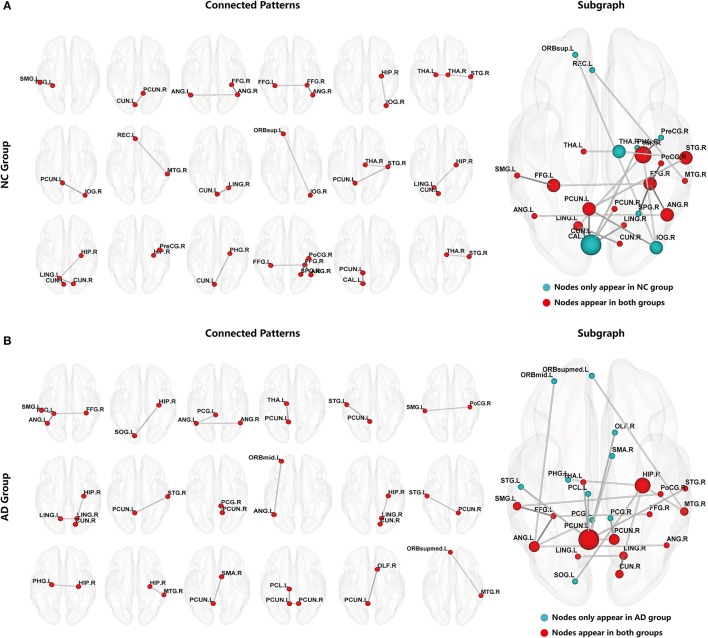
Discriminative subgraphs. **(A)** Denotes normal controls (NC) group, **(B)** Denotes Alzheimer's disease (AD) group. Connected Patterns represent the connectivity pattern of discriminative subgraphs. Subgraph represents that a subgraph was combined discriminative subgraphs within group, where the blue nodes indicate that these nodes are only in the NC group or only in the AD group, and the red nodes indicate that these nodes appear in both NC group and AD group. The nodal size represents the occurrences amount of this node.

After constructing the hyper-network, hyper-edges were extracted as subgraph features from the AD and NC groups. Subgraph features that were repeated within the group were removed to ensure their uniqueness. Then, the FSFS algorithm and the threshold of discriminative score *K* were used to select the most discriminative subgraphs. With the discriminative score *K* threshold set to 0.25, we obtained 18 subgraphs in the NC group and 32 subgraphs in the AD group. To ensure a balanced number of features, the 18 subgraph features with the highest discriminative scores were selected from the AD group. Figure 3 shows the distribution of the discriminative subgraph features in the brain.

To make it easier to analyze the differences between groups, the 18 subgraphs in each group were combined, as shown in Figure 3. The subgraph of group A and the subgraph of group B in Figure 3 were further analyzed. The results showed that the majority of the discriminative regions were those brain regions that appeared together in both groups; however, those that showed significant differences indicated abnormal regions. Figure 3 shows that these abnormal brain regions were mainly distributed in the left PCUN, right HIP, right superior temporal gyrus (STG), right angular gyrus (ANG), right FFG, left FFG, right PCUN, left ANG, left lingual gyrus (LING), right MTG, left SMG, right cuneus (CUN), right LING, left THA, and right postcentral gyrus (PoCG).

**Figure 4:**
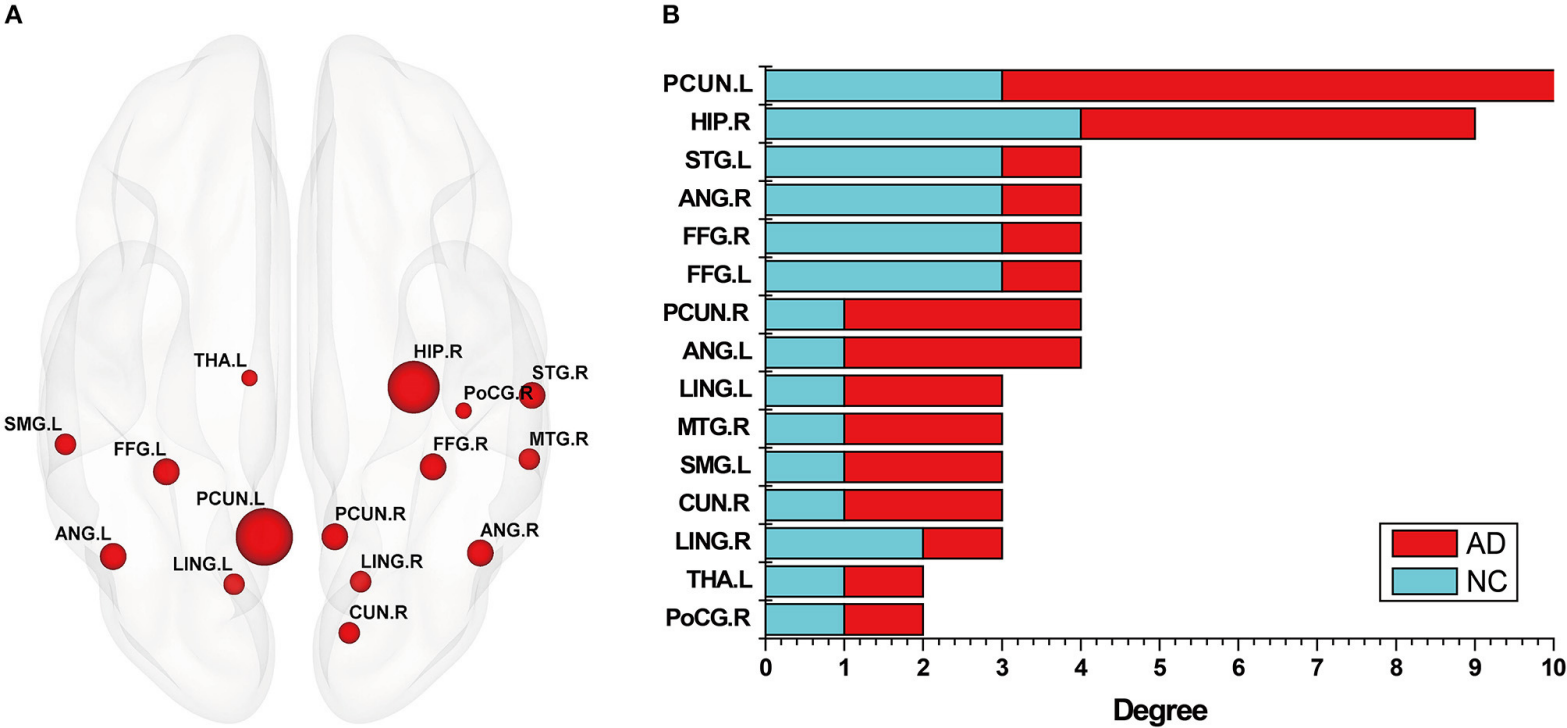
The abnormal brain regions of subgraph feature. **(A)** Denotes these nodes that nodes appear in both normal control (NC) group and Alzheimer's disease (AD) group, where the size of nodes represents the number of occurrences of the node. **(B)** Denotes a statistical chart about the occurrences of these nodes in **(A)**. That is, the occurrences of abnormal brain regions respectively appearing in discriminative subgraphs of NC group and AD group, where red color indicates AD, and blue color indicates NC. Then, ordinate represents these abnormal brain regions, and abscissa represents the occurrences of these brain regions in NC group and AD group, respectively.

In addition, in order to better analyze the abnormal brain regions, and the differences among them in the NC group and AD group, this study examined the distribution of these abnormal regions in the brain. The number of occurrences of abnormal brain regions was summed in the NC and AD groups, and the regions were then sorted according to their sums. Figure 4A shows the distribution of the abnormal regions, and Figure 4B shows the distribution of the sum of occurrences in the NC and AD groups. We counted the sum of occurrences of these abnormal brain regions, and then chose the 10 highest for further analysis: the left PCUN, right HIP, right STG, right ANG, right FFG, left FFG, right PCUN, left ANG, left LING, and right MTG. Table 4 shows the detailed information of the top 10 abnormal regions with significant differences.

**Table 4:** The TOP10 abnormal regions of subgraph feature.

**No**.	**ROI**	**Name**	**Citations**
1	Left	Precuneus	He et al., 2007
2	Right	Hippocampus	Liu et al., 2014
3	Right	Superior temporal gyrus	Solépadullés et al., 2009
4	Right	Angular gyrus	Liu et al., 2014
5	Right	Fusiform gyrus	Yetkin et al., 2006
6	Left	Fusiform gyrus	He et al., 2007
7	Right	Precuneus	He et al., 2007
8	Left	Angular gyrus	Liu et al., 2014
9	Right	Lingual gyrus	He et al., 2007
10	Right	Middle temporal gyrus	Berg et al., 1998

*ROI denotes the region of interest*.

### Classification results

The classification accuracy, specificity, sensitivity, and AUC under the ROC curve were used as a quantitative measure to evaluate the experimental results. To demonstrate the classification performance of the proposed method, we compared the accuracy, sensitivity, and specificity of different classification methods, and analyzed the differences among different network construction methods and feature extraction methods. As can be seen from Table 5, the proposed method performed better than the conventional methods of constructing the functional network by partial correlations or Pearson correlations.

**Table 5:** Classification performance of different methods.

**Method**	**Research**	**Disease**	**Accuracy (%)**	**Sensitivity (%)**	**Specificity (%)**
Partial-network	Rosa et al., 2015	MDD	58.33	53.33	63.33
	Wee et al., 2016	EMCI	62.71	60.00	65.52
	Guo et al., 2013	MDD	86.01	–	–
Pearson-network	Liu F. et al., 2015	MDD	63.00	40.00	83.00
	Wee et al., 2016	EMCI	66.10	76.67	55.17
	Wee et al., 2012	MCI	86.49	–	–
	Wang et al., 2011	AD	–	81.00	73.00
	Chen G. et al., 2011	AD	–	85.00	80.00
Hyper-network	Biao et al., 2014	MCI	94.60	91.70	96.00
	Jie et al., 2016	ADHD	82.90	83.90	86.10
Frequent subgraph	Du et al., 2016	ADHD	94.91	93.22	96.94
	Fei et al., 2014	MCI	97.30	**–**	**–**
Combined multiple features	Zhou et al., 2014	MCI	86.47	–	–
Hyper-network	Subgraph feature	AD	74.80	83.33	67.60
	Brain region feature	AD	83.30	84.21	82.14
	Proposed	AD	91.60	93.50	90.50

*AD represents Alzheimer's disease, MDD represents major depressive disorder, MCI represents mild cognitive impairment, EMCI represents mild cognitive impairment, and ADHD represents attention deficit hyperactivity disorder*.

To accurately compare the different methods of feature extraction, we used the same dataset and constructed the same network, and the brain region features, subgraph features, and multi-features method were used for the classification, respectively. The classification results are shown in Table 5. The experimental results showed that the proposed method's accuracy of 91.60%, specificity of 90.50%, and sensitivity of 93.5% were significantly better than the classification results using only a single feature.

**Figure 5:**
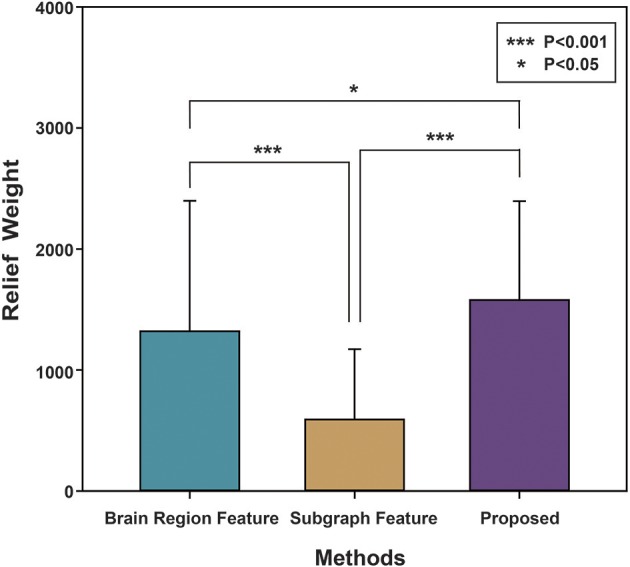
The Relief weight of different methods. The ordinate stands for the Relief weight, and the abscissa denotes different feature extraction methods. Brain region feature represents the Relief weight by using the method based on brain region features. Subgraph feature represents the Relief weight by using the method based on subgraph features. Proposed represents the Relief weight by adopting the method combined subgraph features and brain region features. And then, ^***^indicates that *P*-value obtained by non-arametric permutation test is less than 0.001, and ^*^indicates that *P*-value is less than 0.05.

Finally, the Relief algorithm was used to evaluate the importance of features. The Relief algorithm was first proposed by Kira (Kira and Rendell, 1992) and has been widely applied in selecting features for classification (Rosario and Thangadurai, 2015). As shown in Figure 5, to verify the validity of the proposed method, the brain region feature, subgraph feature, and multi-feature methods were each evaluated by the Relief algorithm. The weight of every feature was obtained according to the correlation between the feature and its class. The greater the relief weight, the stronger the correlation between the feature and the class, indicating that the feature is more important for classification. Figure 5 shows that the relief weight of the multi-feature method was significantly higher than that of the single feature method.

In conclusion, the proposed method of machine learning classification combining multiple features of a hyper-network of fMRI data in AD could be used to effectively classify healthy people and AD patients.

### Effect of regularization parameter λ

The proposed classification model involves setting some parameters, which would be expected to affect the final results. Here, we tested the classification performance with different parameters, including the regularization parameter λ of the sparse target optimization function, the threshold of the discriminative score *K*, and the optimal weighting parameter α^*i*^, and attempted to determine the optimal parameter settings.

**Figure 6:**
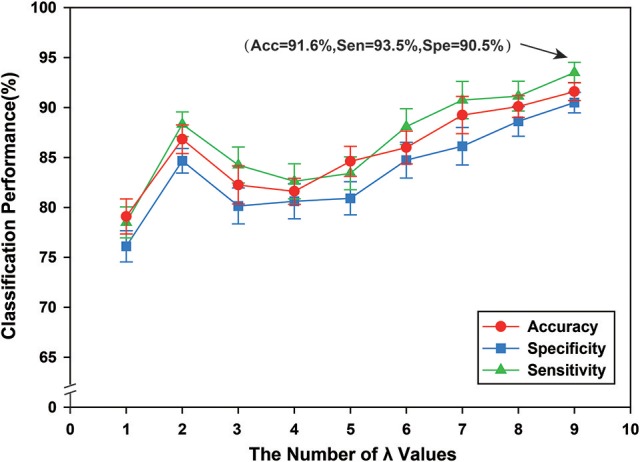
The classification performance of different regularization parameters λ. The ordinate indicates accuracy, specificity and sensibility of this method, and the abscissa denotes nine groups of different regularization parameter λ, where 1 represents that λ value is {0.1}, and 2 denotes that λ value is {0.1, 0.2}, and 3 denotes that λ value is {0.1, 0.2, 0.3}, …, 9 represents that {0.1, 0.2, …, 0.9}. Thus, when λ is {0.1, 0.2, …, 0.9}, better classification performance can be obtained, including that accuracy is 91.60%, and specificity is 93.50%, and then sensibility is 90.50%.

We constructed the hyper-networks by adopting a sparse representation method, where λ indicates a regularization parameter for controlling the sparsity of the network (λ > 0). By setting different values of λ within a required range, we obtained an array of hyper-edges. To research the classification performance of this method with different λ values, nine groups of different λ values were tested, {0.1}, {0.1, 0.2}, {0.1, 0.2, 0.3}, …, {0.1, 0.2, …, 0.9}. The classification results indicated that a greater number of λ values corresponded with better classification performance. In a previous study (Jie et al., 2016), the λ value was set to {0.1, 0.2, …, 0.9}, as shown in Figure 6, and was confirmed experimentally. Therefore, in this study, λ was set to {0.1, 0.2, …, 0.9}. Figure 6 shows the classification performance under different regularization parameters.

### Effect of discriminative score threshold *K*

**Figure 7:**
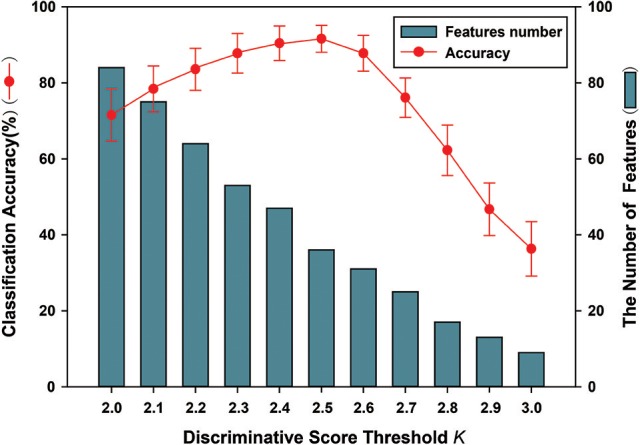
The classification accuracy and the number of features under different discriminative score threshold *K*. The ordinate indicates accuracy of this method, and the abscissa denotes different discriminative score threshold *K*, ranging from 2.0 to 3.0 at a step size of 0.1. As shown in the figure, when *K* = 0.25, the number of features is 36. Meanwhile, better classification accuracy was obtained; including that accuracy is 91.6%.

The FSFS algorithm was adopted to select discriminative subgraphs. Because of the excessive number of selected subgraphs, a threshold value was set (the discriminative score threshold *K*). The other parameters were controlled to select a more accurate discriminative score threshold *K*. The threshold *K* ranged from 0.20 to 0.30 and the interval was 0.01. Figure 7 shows the classification accuracy and the number of features under different discriminative score threshold *K*-values. The experimental results showed that when the discriminative score threshold *K* = 0.25, the number of features was 36, and the classification accuracy was optimal. One potential explanation is that when the threshold was too small, features that contributed little to the classification were also chosen, but when it was too large, features that made large contributions were removed, leading to lower classification accuracy in both cases.

### Effect of optimal weighting parameter α_i_

**Figure 8:**
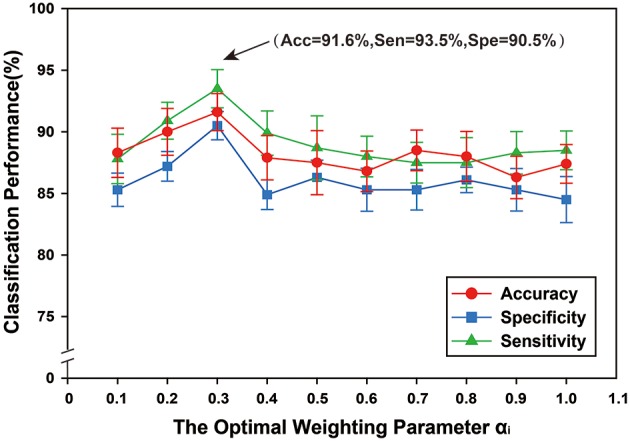
The classification performance of different optimal weighting parameters α_*i*_. The ordinate indicates accuracy, specificity and sensibility of this method, and the abscissa denotes different optimal weighting parameter α^*i*^, ranging from 0 to 1 at a step size of 0.1, and $\overset{M}{\underset{i=1}{\sum }}\alpha_{i}=1$. As shown in the figure, when α^*i*^ = 0.3, better classification performance was obtained, including that accuracy is 91.60%, and specificity is 93.50%, and then sensibility is 90.50%.

A multi-kernel SVM was used for classification, which involved finding the optimal weighting parameter α_*i*_. To examine the effects of different values of α_*i*_ on classification performance, the range was set from 0 to 1, with a step size of 0.1 and $\overset{M}{\underset{i=1}{\sum }}\alpha_{i}=1.$ Figure 8 shows the classification performance under different optimal weighting parameters α_*i*_. The best classification performance was obtained when α_*i*_ = 0.3, with accuracy of 91.60%, sensitivity of 93.50%, and specificity of 90.5%. The experimental results showed that different values of optimal weighting parameters α_*i*_ influenced the classification results.

## Discussion

In this study, we proposed a method of machine learning classification combining multiple features of a hyper-network of fMRI data in AD. Hyper-networks were constructed on the AD dataset to analyze the interactions among multiple brain regions. Then, two types of features were used for feature extraction and selection: brain region features were selected using a non-parametric test method, and subgraph features were selected using the FSFS algorithm. Finally, two types of kernel (vector kernel and graphkernel) were fused, and a multi-kernel SVM classifier was used for classification. The experimental results verified the validity of the proposed method.

### The most discriminative brain regions using brain region features

Two methods were used to discriminate significantly abnormal brain regions between groups. The results using only brain region features showed 13 abnormal regions, as shown in brain region features of experiments and results section. Many previous researches have found that these brain regions are abnormal in AD patients. Specifically, the posterior cingulate cortex (PCC) is mainly involved in episodic memory and short-term memory processing (Gusnard and Raichle, 2001; Buckner et al., 2008) and is a critical region in human brain structural and functional networks (Greicius et al., 2004; Cavanna and Trimble, 2006; Zhang et al., 2009; Binnewijzend et al., 2012). Studies have shown that the PCC is one of the most robust brain regions in the resting state. The PCUN is also an important component of the default mode network, and is closely related to the extraction of episodic memory (Fransson and Marrelec, 2008). Using r-fMRI, several recent studies have suggested that the PCC/PCu exhibits reduced regional activity in AD patients (He et al., 2007). In addition, using resting-state fMRI to measure the amplitude of low-frequency fluctuations (ALFF) of intrinsic brain activity in 23 patients with moderate AD and 27 age- and gender-matched healthy controls, Liu et al. (2014) found that AD patients also showed increased ALFF in the bilateral Hip/PHG. The Hip/PHG is considered to be critical to memory function. Compared with normal controls, the AD patients showed decreased ALFF values in the bilateral PCC/PCu, MTG, and STG. Yetkin et al. (2006) proved that AD patients showed more activation than controls in the right MFG, left ITG, left THA, and right PUT and so on. Wang et al. (2011) used resting-state functional MRI to investigate spatial patterns of spontaneous brain activity in 22 healthy elderly subjects, 16 MCI, and 16 AD patients. The results showed that ALFF differences between AD patients and healthy elderly subjects were mainly found in the bilateral PHG/Hip, bilateral SMA, and left FFG. The results obtained in this study are consistent with those of previous studies.

### The most discriminative brain regions using subgraph features

The results using only subgraph features showed that the abnormal brain regions included the left PCUN, right HIP, right STG, right ANG, right FFG, left FFG, right PCUN, left ANG, left LING, right MTG, left SMG, right CUN, right LING, left THA, and right PoCG. These abnormal brain regions have been shown to be associated with AD in previous studies. Both structural MRI and resting-state fMRI scans were collected from 14 AD subjects and 14 age-matched normal controls. He et al. (2007) found that regional coherence was significantly decreased in the PCUN in the AD patients compared with the normal controls. Recent functional imaging studies have indicated that the pathophysiology of AD may be associated with changes in spontaneous low-frequency (<0.08 Hz) blood oxygenation level dependent fluctuations (LFBF) measured during a resting state (He et al., 2007). He et al. also found that AD patients showed increased LFBF coherence in the bilateral CUN, right LING, and left FFG. Neuropathological studies indicate that brain lesions are already present in the inferior parietal lobule (IPL) (including the ANG and left SMG) in incipient AD, although they are observed less frequently than in medial temporal areas (Berg et al., 1998; Markesbery et al., 2006; Haroutunian et al., 2007). Liu et al. (2014) found that AD patients also showed increased ALFF in the IPL. Yetkin et al. (2006) evaluated brain activation in patients with probable AD, MCI, and controls while performing a working memory task. The AD group showed more right FFG and left THA activation than the control group. In this study, the right HIP (Liu et al., 2014) and right STG (Solépadullés et al., 2009) are consistent with previous studies.

### Classification performance

Conventional methods of constructing functional networks cannot reflect the interactions among multiple brain regions and thus ignore the higher-order information among them. To study the complex interaction information among multiple brain regions, Jie et al. (2016) proposed to construct a hyper-network model. In the Jie et al. study, the local brain region properties were extracted from the hyper-network as features, and the most discriminative features were selected. Finally, the multi-kernel SVM was adopted for classification. The construction of the hyper-networks enabled us to identify the interaction information among brain regions. In addition, to show that the classification method based on subgraph features can better capture the topological information among brain regions, Fei et al. (2014) adopted frequent subgraph mining technology to mine frequent sub-networks in an MCI dataset, then used a discriminative subgraph mining algorithm to mine discriminative sub-networks. Finally, they used SVM based on a graph kernel for the classification. Du et al. (2016) used the frequent subgraph mining technique to mine frequent sub-networks in an ADHD dataset, then the FSFS method to select the sub-networks, and graph kernel principal component analysis to extract the features. Finally, SVM was used to classify the data. Wang et al. (Zhou et al., 2014) adopted the same technology to mine frequent sub-networks in an MCI dataset, and then combined the discriminative sub-networks with conventional quantitative properties to select features. Finally, they used multi-kernel SVM for classification. The above results show that classification methods based on subgraph features can effectively improve classification performance.

**Figure 9:**
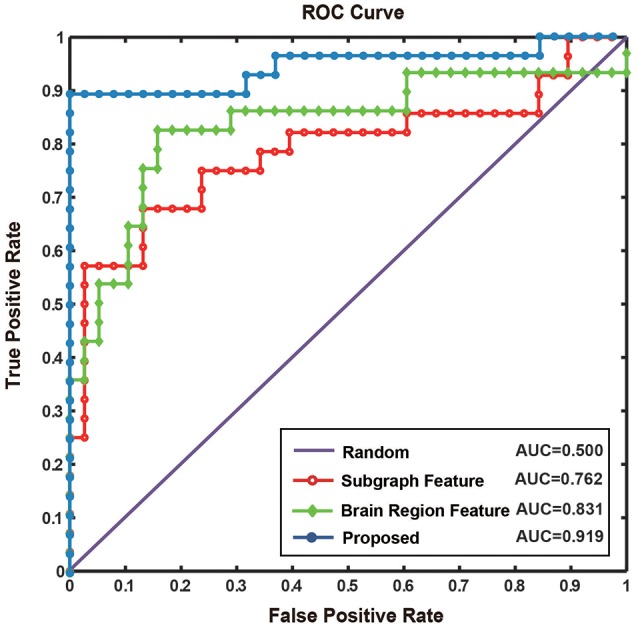
The ROC curve of different methods. Random represents the ROC curve by randomly selecting sample, where AUC value is 0.500. Subgraph feature represents the ROC curve by adopting the method based on subgraph features, where AUC value is 0.762. Brain region feature represents the ROC curve by adopting the method based on brain region features, where AUC value is 0.831. Proposed represents the ROC curve by adopting the method combined subgraph features and brain region features, where AUC value reach to 0.919.

The results of this study were compared with those obtained by conventional methods of functional connectivity network construction based on partial or Pearson correlations (Table 5). The results showed that the proposed method does not just identify the interactive information between brain regions, but can effectively represent the higher-order information among them. In addition, Jie et al. (2016) experimented in the MCI dataset. In comparison, the classification performance of the proposed method was similar and the difference might have been due to the use of different datasets. The same method using different datasets may obtain different classification results, and different methods also differ in the way they construct the network and extract and classify features. To accurately compare the different methods of feature extraction, we used the same dataset and constructed the same hyper-network, and compared the classification results using the brain region feature method, subgraph feature method, and multi-feature method, respectively (Table 5). The diagnostic accuracy of the multi-feature method was 8.3% better than that obtained using only single features. Furthermore, Figure 9 shows the ROC curves for the different classification methods. The AUC value was 0.762 for the subgraph feature method and 0.831 for the brain region feature method, compared with 0.919 for the multi-feature classification method, an increase of at least 0.088. The results show that the proposed classification method combining subgraph features and brain region features preserved not only the global topological information of the brain region, but also the sensitivity to change in a single brain region. The multi-feature classification method can effectively improve the diagnosis accuracy of AD.

The Relief algorithm was used to verify the importance of the underlying features for classification accuracy, with significance analyzed by a non-parametric permutation test. As shown in Figure 5, the average Relief weight of the multi-feature method was significantly higher than that of the single-feature method, indicating that the multi-feature method was better for assessing the importance of features. However, the underlying reason was that the multi-feature based method effectively fused two different yet complementary interaction information: brain region features and subgraph features. Therefore, it not only reflected the information from a single brain region, but also captured the global topological information among brain regions. All of the above experimental results demonstrate the validity of the proposed method.

### Features selection

The current findings demonstrated that the multi-feature combination method effectively integrated multiple network properties, further improving classification performance. The relief analysis method was performed to evaluate the contributions of selected features during the classification process. The relief weight obtained with multiple combined features was significantly greater than the weight obtained when only brain region features or subgraph features were adopted. Regarding the underlying mechanisms, this is likely to be because adopting multiple features can integrate complementary network information, combining local brain region features and subgraph features, thus further improving classification accuracy. Some studies have also demonstrated that multiple features can effectively combine multiple different complementary network properties for classification (Jie et al., 2014; Zhou et al., 2014). Global network topology information will be lost only from the perspective of the brain region features. In addition, subgraph features can also result in the loss of sensitivity of a single brain region.

### Regularization parameter λ

Previous researches had demonstrated that the parameter λ had a great effect on the hyper-network structure. The parameter λ determined the sparsity and scale of network regions. If λ was too small, the network would be too coarse and involve much noise; if λ was too large, the network would be too sparse (Lv et al., 2015). Besides, it was found that the reliability of network structure, especial modularity, was sensitive to the sparsity which was controlled by λ (Xuan and Wang, 2015). Furthermore, the parameter λ also impacted on the classification performance. The ultimate classification accuracy was extremely sensitive to the network model parameters, especial λ (Qiao et al., 2016). As the authors known there was no golden criterion for selection of λ. How to find a suitable λ was important for the construction of hyper-network and classifier. Some optimization methods were proposed. Qiao et al. conducted parameter selection in a large range by computing the classification accuracy based on leave one out test on all the subjects, choosing the corresponding parametric of the best classification accuracy (Qiao et al., 2016). Xuan et al. chose the parameter λ by computing the value of intra-class correlation coefficient (Xuan and Wang, 2015), which could describe the reliability of network structure (Braun et al., 2012). However, It was found that it was hard to achieve a high reliable network structure by setting a single λ. The research showed that the network achieved a relatively high reliability only when λ took 0.01 (it was very close to 0, which suggested that almost all the nodes in the network were connected in the given hyper-edge). In other cases, it performed moderately (Xuan and Wang, 2015). Multi-level λ setting method was proposed (Jie et al., 2016). Different from single λ setting, multi-level λ setting method set a combination of several λ which provided more network structure topology information than the former method. Multi-level λ setting method could avoid the arbitrary decision of single λ setting method and reduce the influence of the low reliability caused by single network structure.

How to get the most optimizing combination of λ values was one of the important thing in the multi-level λ setting method. Enumeration method was not suitable because of the huge computation consumption caused by the large amount of random combinations. For a nine intervals setting which was adopted in the current study, there were 511 different combinations in total (${\text{C}}_{9}^{1}+{\text{C}}_{9}^{2}+{\text{C}}_{9}^{3}+{\text{C}}_{9}^{4}+{\text{C}}_{9}^{5}+{\text{C}}_{9}^{6}+{\text{C}}_{9}^{7}+{\text{C}}_{9}^{8}+{\text{C}}_{9}^{9}=511$). More intervals could result in more combinations. In the current study, a series of serial ascending order combinations was adopted, embodying as {0.1}, {0.1, 0.2}, …, {0.1, 0.2, …, 0.9} (nine combinations in total). The method remained small λ values in the combinations as many as possible that means more nodes were connected in the constructed hyper-edges. It was thought that the hyper-edges with many nodes could describe the underlying relationship among several nodes. Reverse order combination was not taken into account because many large λ values was remained in the combinations. Strict λ setting could result in a few nodes in the constructed hyper-edges. In our experiments, it was found that almost all the hyper-edges connected only two nodes when λ was 0.9. It suggested the hyper-network had degenerated into the conventional network. Admittedly, ascending order method was still arbitrary. It was one of the limitations of the current study. A feasible optimizing combination selection method should be researched in the future.

To characterize multi-level relationships within multiple brain regions, it is necessary to set a range of different λ values. The more λ values, the more interaction information among multiple brain regions contained in the hyper-edges. In the current study, we set nine groups of different λ values and the classification results showed that a greater number of λ values corresponded to better classification performance. This result suggests that when a hyper-edge contains more multi-level interaction information, the hyper-network reflects greater structural differences among the different samples. These structural differences could be embodied both by node metrics and hyper-edge connection patterns. Therefore, the advantages of multiple sparse levels indicates the superiority of hyper-networks compared with conventional simple networks.

### Limitations

We proposed a method of machine learning classification combining multiple features of a hyper-network of fMRI data in AD, which could be used to effectively classify normal controls and AD patients. However, there were some limitations. A sparse linear regression model was used to construct the hyper-networks. However, when constructing the hyper-edges, for a chosen brain region, if the pairwise correlations between other brain regions were very high, then this method tended to select only one region with a grouping effect from the group, but did not care which one was selected. It is possible that some related brain regions were not selected, which means the grouping effect information could not be explained. Constructing the hyper-network based on sparse representation confirmed the stability of constructed hyper-edges, which is also an important step. To address this limitation in future studies, we plan to adopt other effective methods, such as the robust least absolute shrinkage and selection operator (LASSO) (Xu et al., 2008) and group LASSO (Yuan and Lin, 2006).

## Repeatability verification

To further verify the repeatability of the proposed method, we tested it with the public Alzheimer's Disease Neuroimaging Initiative (ANDI) data set. The data set included data from 94 subjects, including 33 early mild cognitive impairment (EMCI) patients, 32 late mild cognitive impairment (LMCI) patients and 29 AD patients. There were no significant differences in gender or age among the four groups, but mini-mental state examination (MMSE) scores were significantly different among the groups. Demographics and clinical characteristics of the subjects are listed in Table 6.

**Table 6:** Demographics and clinical characteristics of the subjects.

	**EMCI**	**LMCI**	**AD**	***P*-value**
Number(n)	33	32	29	–
Female/male(n)	16/17	13/19	18/11	0.53^a^
Age (mean ± SD, year)	72.0 ± 6.0	72.6 ± 8.3	72.3 ± 7.4	0.48^a^
MMSE	27.6 ± 2.1	26.9 ± 2.7	21.0 ± 3.6	<0.0001^b^

*EMCI, early mild cognitive impairment; LMCI, late mild cognitive impairment; AD, Alzheimer's disease; MMSE, Mini-Mental State Examination*.

a *chi-square test*.

b *one-way analysis of variance tests*.

We adopted the same method as described above. First, a sparse linear model was used to construct the hyper-network with the EMCI data set. Local brain region properties (HCC1, HCC2, and HCC3) and subgraphs were then extracted as features. Finally, two different features were combined, and multi-kernel SVM was adopted to perform classification. Ten-fold cross-validation was repeated 100 times. The experimental protocol was repeated with both the LMCI and AD data sets.

The local brain region properties HCC1, HCC2, and HCC3 were further analyzed, and significant differences (*p* < 0.05, FDR correction) in abnormal brain regions were selected for the three groups. Tables 7–9 list the abnormal brain regions between NC group and EMCI group, LMCI group, AD group respectively. As above, the FSFS algorithm was adopted to select discriminative subgraphs in the three data sets. To achieve a similar number of discriminative subgraph features to that in our previous study, the discriminative score K threshold was set at 0.2. We obtained 24 discriminative subgraphs between the NC and EMCI groups, and 30 discriminative subgraphs between the NC and LMCI groups, and 32 discriminative subgraphs between the NC and AD groups. We further analyzed the subgraph patterns for each group pair, revealing the abnormal brain regions between them. Detailed results are shown in the Supplemental Figure S1.

**Table 7:** The abnormal brain regions and significance of brain region feature between the normal control group and the early mild cognitive impairment group.

**No**	**ROI**	**Name**	***P*****-values**		
			**HCC_1_**	**HCC_2_**	**HCC_3_**
1	Left	Superior frontal gyrus, medial	**0.0396**	0.5082	0.6102
2	Right	Middle temporal gyrus	0.7149	**0.0442**	0.9990
3	Right	Olfactory cortex	0.1616	**0.0396**	0.6522
4	Right	Temporal pole: superior temporal gyrus	0.3013	**0.0050**	0.2474
5	Right	Parahippocampal gyrus	0.4322	**0.0352**	**0.0272**
6	Right	Postcentral gyrus	0.2351	0.1286	**0.0352**
7	Right	Angular gyrus	0.6102	0.1363	**0.0129**

*ROI denotes the reigon of interest. Bold indicates significance p < 0.05*.

**Table 8:** The abnormal brain regions and significance of brain region feature between the normal control group and the late mild cogniticve impairment group.

**No**	**ROI**	**Name**	***P*****-values**		
			**HCC_1_**	**HCC_2_**	**HCC_3_**
1	Left	Posterior cingulate gyrus	**0.0371**	0.8474	0.3563
2	Left	Middle frontal gyrus, orbital part	0.8915	**0.0134**	0.9569
3	Left	Insula	0.7154	**0.0338**	0.6870
4	Right	Amygdala	0.5039	**0.0134**	0.6727
5	Right	Temporal pole: middle temporal gyrus	0.9109	**0.0291**	0.3563
6	Right	Rolandic operculum	0.6727	0.8474	**0.0353**
7	Left	Supplementary motor area	0.6727	0.1936	**0.0250**
8	Left	Anterior cingulate and paracingulate gyri	0.1487	0.1668	**0.0405**
9	Right	Calcarine fissure and surrounding cortex	0.3675	0.9015	**0.0337**
10	Right	Postcentral gyrus	0.1376	0.1606	**0.0216**

*ROI denotes the reigon of interest. Bold indicates significance p < 0.05*.

**Table 9:** The abnormal brain regions and significance of brain region feature between the normal control group and the Alzheimer's disease group.

**No**	**ROI**	**Name**	***P*****-values**		
			**HCC_1_**	**HCC_2_**	**HCC_3_**
1	Right	Calcarine fissure and surrounding cortex	**0.0060**	0.9512	0.9580
2	Right	Cuneus	**0.0058**	0.9641	0.7833
3	Left	Lingual gyrus	**0.0067**	0.4542	0.2203
4	Left	Fusiform gyrus	**0.0155**	0.8045	0.5478
5	Right	Superior parietal gyrus	**0.0343**	0.1704	**0.0390**
6	Left	Thalamus	**0.0138**	0.6394	0.8114
7	Right	Inferior frontal gyrus, triangular part	0.5780	**0.0301**	0.6009
8	Right	Amygdala	0.1272	**0.0160**	0.5629
9	Right	Temporal pole: middle temporal gyrus	0.2514	**0.0317**	0.2289
10	Left	Inferior frontal gyrus, opercular part	0.6086	0.2079	**0.0334**
11	Right	Olfactory cortex	0.8695	0.2908	**0.0179**
12	Left	Superior occipital gyrus	0.2246	0.0912	**0.0325**
13	Right	Postcentral gyrus	0.5553	**0.0189**	**0.0074**
14	Left	Inferior parietal, but supramarginal and angular gyri	0.0642	0.2706	**0.0361**

*ROI denotes the region of interest. Bold indicates significance p < 0.05*.

We compared the abnormal regions of subgraph features of AD patients in our collected dataset and ADNI dataset. It was found that there were many overlapped abnormal regions between two datasets (Table 10). In the top 10 abnormal regions, there were seven regions found both in our collected dataset and ADNI dataset, including right hippocampus, right lingual gyrus, right fusiform gyrus, left fusiform gyrus, left precuneus, left angular gyrus and right superior temporal gyrus. In addition, we compared the abnormal brain regions of the EMCI, LMCI, and AD groups in ADNI dataset (Table 11). The results showed that there were a large number of overlapped abnormal brain regions between EMCI and LMCI, which included right dorsolateral superior frontal gyrus, left middle frontal gyrus, right middle frontal gyrus, left medial superior frontal gyrus, right medial superior frontal gyrus, left insula, left superior occipital gyrus and left parahippocampal gyrus. The above overlapped regions showed that the subgraph features had good repeatability and stability. Analysis showed that many abnormal brain regions of EMCI and LMCI groups were located in the frontal lobe and limbic system. It was noted that only one overlapped abnormal brain region (right hippocampus) was found between LMCI and AD groups. The results indicated that there were obvious differences in the abnormal brain regions obtained by subgraph features of different diseases.

**Table 10:** Top 10 abnormal brain regions of subgraph features of the Alzheimer's disease patients in our collected dataset and ADNI dataset.

**No**	**Our Collected Dataset**		**Citations**	**ADNI Dataset**		**Citations**
1	Left	Precuneus^+^	He et al., 2007	Right	Hippocampus^+^	Liu et al., 2014
2	Right	Hippocampus^+^	Liu et al., 2014	Right	Lingual gyrus^+^	He et al., 2007
3	Right	Superior temporal gyrus^+^	Solépadullés et al., 2009	Right	Fusiform gyrus^+^	Yetkin et al., 2006
4	Right	Angular gyrus	Liu et al., 2014	Left	Lingual gyrus	He et al., 2007
5	Right	Fusiform gyrus^+^	Yetkin et al., 2006	Left	Fusiform gyrus^+^	He et al., 2007
6	Left	Fusiform gyrus^+^	He et al., 2007	Left	Precuneus^+^	He et al., 2007
7	Right	Precuneus	He et al., 2007	Left	Posterior cingulate gyrus	He et al., 2007
8	Left	Angular gyrus^+^	Liu et al., 2014	Left	Angular gyrus^+^	Liu et al., 2014
9	Right	Lingual gyrus^+^	He et al., 2007	Right	Superior temporal gyrus^+^	Solépadullés et al., 2009
10	Right	Middle temporal gyrus	Berg et al., 1998	Right	Cuneus	He et al., 2007

+ *Indicates the abnormal brain region appears in both datasets. ADNI, Alzheimer's Disease Neuroimaging Initiative*.

**Table 11:** Top 10 abnormal brain regions of subgraph features in the early mild cognitive impairment group, the late mild cognitive impairment group and Alzheimer's disease group in ADNI dataset.

**No**	**EMCI Group**		**Citations**	**LMCI Group**		**Citations**	**AD Group**	
1	Right	Superior frontal gyrus, dorsolateral^×^	Yetkin et al., 2006	Right	Superior frontal gyrus, dorsolateral^×^	Yetkin et al., 2006	Right	Hippocampus^*^
2	Left	Middle frontal gyrus ^×^	Zhao et al., 2015	Left	Superior frontal gyrus, medial^×^	Yetkin et al., 2006	Right	Lingual gyrus
3	Right	Middle frontal gyrus^×^	Zhao et al., 2015	Left	Parahippocampal gyrus^×^	Liu J. et al., 2015	Right	Fusiform gyrus
4	Right	Olfactory cortex	Zhao et al., 2015	Right	Precentral gyrus	Devanand et al., 2006	Left	Lingual gyrus
5	Left	Superior frontal gyrus, medial^×^	Yetkin et al., 2006	Left	Middle frontal gyrus^×^	Zhao et al., 2015	Left	Fusiform gyrus
6	Right	Superior frontal gyrus, medial^×^	Yetkin et al., 2006	Right	Middle frontal gyrus^×^	Zhao et al., 2015	Left	Precuneus
7	Left	Insula^×^	Devanand et al., 2006	Right	Superior frontal gyrus, medial^×^	Yetkin et al., 2006	Left	Posterior cingulate gyrus
8	Left	Superior occipital gyrus^×^	Morgen et al., 2013	Left	Insula^×^	Devanand et al., 2006	Left	Angular gyrus
9	Left	Parahippocampal gyrus^×^	Liu J. et al., 2015	Left	Superior occipital gyrus^×^	Morgen et al., 2013	Right	Superior temporal gyrus
10	Left	Fusiform gyrus	Lim et al., 2011	Right	Hippocampus^*^	Morgen et al., 2013	Right	Cuneus

× *Indicates the abnormal brain region appears in both EMCI and LMCI groups*.

* *Indicates the abnormal brain region appears in both LMCI and AD groups*.

*EMCI, early mild cognitive impairment; LMCI, late mild cognitive impairment; AD, Alzheimer's disease; ADNI, Alzheimer's Disease Neuroimaging Initiative. The citations of AD group are shown in Table 10*.

Meanwhile, we compared the abnormal regions of brain region features of AD patients in our collected dataset and ADNI dataset. Differentiating from subgraph features, the results of brain region features showed that there were only two overlapped abnormal brain regions, including left fusiform gyrus and left thalamus (Table 12). In addition, after compared among EMCI, LMCI and AD groups in ADNI dataset, the results of abnormal regions showed a great deal of difference (Table 13). There was only one brain region (right postcentral gyrus) appearing in the all three groups. Only one brain region (right olfactory cortex) overlapped in EMCI and AD groups and three brain regions (right amygdala, right temporal pole: middle temporal gyrus and right calcarine fissure and surrounding cortex) overlapped in LMCI and AD groups. There was not any region overlapped in EMCI and LMCI groups. The results showed that, compared the subgraph features, the brain region features were not stable. The abnormal brain regions obtained by brain region features were significantly different in different datasets or different diseases. Furthermore, the contrast analysis found a result consistent with subgraph features. The result showed that the abnormal brain regions mainly were located in the frontal lobe and limbic lobe in EMCI and LMCI groups (Pennanen et al., 2005; Whitwell et al., 2008; Schroeter et al., 2009; Wang et al., 2011).

**Table 12:** The abnormal brain regions of brain region features of the Alzheimer's disease patients in our collected dataset and ADNI dataset.

**No**	**Our Collected Dataset**		**Citations**	**ADIN Dataset**		**Citations**
1	Right	Middle frontal gyrus	Wang et al., 2011	Right	Calcarine fissure and surrounding cortex	Hampel et al., 2002
2	Left	Inferior temporal gyrus	Wang et al., 2011	Right	Cuneus	He et al., 2007
3	Right	Posterior cingulate gyrus	He et al., 2007	Left	Lingual gyrus	He et al., 2007
4	Left	Supplementary motor area	Wang et al., 2011	Left	Fusiform gyrus^+^	He et al., 2007
5	Right	Parahippocampal gyrus	Wang et al., 2011	Right	Superior parietal gyrus	Zhou and Jin, 2008
6	Right	Inferior temporal gyrus	Wang et al., 2011	Left	Thalamus^+^	Solépadullés et al., 2009
7	Right	Precuneus	He et al., 2007	Right	Inferior frontal gyrus, triangular part	He et al., 2007
8	Left	Fusiform gyrus^+^	He et al., 2007	Right	Amygdala	Hampel et al., 2002
9	Left	Supramarginal gyrus	Grady et al., 2003	Right	Temporal pole: middle temporal gyrus	Zhou and Jin, 2008
10	Right	Hippocampus	Liu et al., 2014	Left	Inferior frontal gyrus, opercular part	He et al., 2007
11	Right	Lenticular nucleus, putamen	De-Jong et al., 2008	Right	Olfactory cortex	Vasavada et al., 2015
12	Left	Thalamus^+^	Solépadullés et al., 2009	Left	Superior occipital gyrus	Grady et al., 2003
13	Left	Middle temporal gyrus	Grady et al., 2003	Right	Postcentral gyrus	Wang et al., 2011
14		–	–	Left	Inferior parietal, but supramarginal and angular gyri	Grady et al., 2003

+ *indicates the abnormal brain region appears in both groups. ADNI, Alzheimer's Disease Neuroimaging Initiative*.

**Table 13:** The abnormal brain regions differences of brain region feature among the early mild cognitive impairment group, the late mild cognitive impairment group and Alzheimer's disease group in ADNI dataset.

**No**	**EMCI Group**		**Citations**	**LMCI Group**		**Citations**	**AD Group**	
1	Left	Superior frontal gyrus, medial	Yetkin et al., 2006	Left	Posterior cingulate gyrus	Zhao et al., 2015	Right	Calcarine fissure and surrounding cortex^*^
2	Right	Middle frontal gyrus	Zhao et al., 2015	Left	Middle frontal gyrus, orbital part	Zhao et al., 2015	Right	Cuneus
3	Right	Olfactory cortex^Δ^	Zhao et al., 2015	Left	Insula	Devanand et al., 2006	Left	Lingual gyrus
4	Right	Temporal pole: superior temporal gyrus	Fei et al., 2014	Right	Amygdala^*^	Yao et al., 2014	Left	Fusiform gyrus
5	Right	Parahippocampal gyrus	Liu J. et al., 2015	Right	Temporal pole: middle temporal gyrus^*^	Morgen et al., 2013	Right	Superior parietal gyrus
6	Right	Postcentral gyrus^++^	Devanand et al., 2006	Right	Rolandic operculum	Fei et al., 2014	Left	Thalamus
7	Right		Liu J. et al., 2015	Left	Supplementary motor area	Poettrich et al., 2009	Right	Inferior frontal gyrus, triangular part
8	–		–	Left	Anterior cingulate and paracingulate gyri	Zhao et al., 2015	Right	Amygdala^*^
9	–		–	Right	Calcarine fissure and surrounding cortex^*^	Zhao et al., 2015	Right	Temporal pole: middle temporal gyrus^*^
10	–		–	Right	Postcentral gyrus^++^	Devanand et al., 2006	Left	Inferior frontal gyrus, opercular part
11	–		–		–	–	Right	Olfactory cortex^Δ^
12	–		–		–	–	Left	Superior occipital gyrus
13	–		–		–	–	Right	Postcentral gyrus^++^
14	–		–		–	–	Left	Inferior parietal, but supramarginal and angular gyri

* *Indicates the abnormal brain region appears in both LMCI and AD groups*.

Δ *Indicates the abnormal brain region appears in both EMCI and AD groups*.

++ *Indicates the abnormal brain region appears in all the three groups. EMCI, early mild cognitive impairment; LMCI, late mild cognitive impairment; AD, Alzheimer's disease; ADNI, Alzheimer's Disease Neuroimaging Initiative. The citations of AD group are shown in Table 12*.

In this paper, the subgraph features and the brain region features were used as the features of classification, which described the different network properties from different perspectives. Subgraph features were represented as connected patterns while brain region features were represented as quantifiable values. Compared with brain region features, subgraph features showed better repeatability and stability. To be specific, there were more overlapped abnormal brain regions of subgraph features, no matter in different datasets (AD group in our collected dataset vs. AD group in ADNI dataset) or different diseases (EMCI group in ADNI dataset vs. LMCI group in ADNI dataset). The result suggested that the differences of network structure, which were embodied by connected pattern, were not susceptible to the different datasets. It should be noted that the characteristic of subgraph features also implied that it was insensitive to the changes of samples. On the contrary, the brain region features were sensitive to the changes of samples. Different datasets showed significant differences in abnormal brain regions. Therefore, the abnormal brain regions obtained by brain region features in a dataset were difficult to apply to other datasets. Although the repeatability of the brain region features was not strong, the quantifiable local property could capture the specific inter-group differences. These differences could distinguish between the given groups, despite they were not repeatable in other datasets. The direct evidence of this conclusion was that the classification accuracy of brain region features was higher than that of subgraph features in the both datasets (Tables 5, 14).

**Table 14:** Classification performance of different methods.

**Method**	**Research**	**Diseases**	**Accuracy (%)**	**Sensitivity (%)**	**Specificity (%)**
Hyper-network	Subgraph feature	EMCI	64.90	70.10	61.60
	Brain region feature	EMCI	69.21	73.27	65.67
	proposed	EMCI	72.80	78.25	67.13
	Subgraph feature	LMCI	71.10	76.33	66.95
	Brain region feature	LMCI	74.33	79.21	69.00
	proposed	LMCI	78.63	82.54	72.18
	Subgraph feature	AD	75.60	78.33	71.05
	Brain region feature	AD	80.23	81.25	77.90
	proposed	AD	88.91	91.73	85.66

*EMCI, early mild cognitive impairment; LMCI, late mild cognitive impairment; AD, Alzheimer's disease*.

We performed classification in the three different group pairs separately, including NC vs. EMCI, NC vs. LMCI and NC vs. AD. In addition, to compare the different feature extraction methods, we also performed classification using only brain region features and subgraph features (Table 14). In the three different group pairs, the multi-feature method consistently showed better classification performance than the single feature method. This result is consistent with the findings of our previous study. In addition, the classification accuracy between the NC and AD groups reached 88.91%, which was closed to the results of our self-collection data set (91.60%). These findings suggest that our proposed method is stable and repeatable.

**Figure 10:**
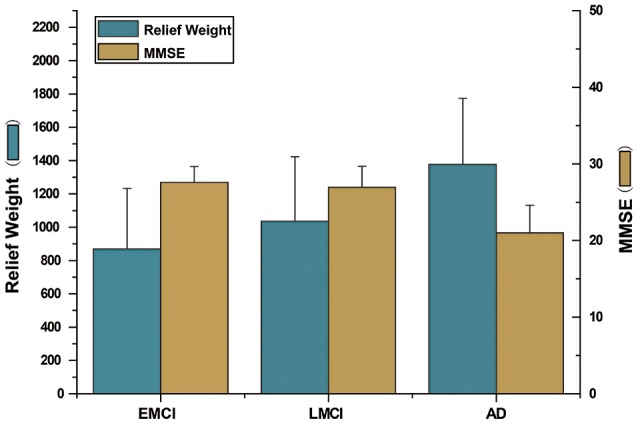
The illustration of relief weight and MMSE score. The left ordinate indicates relief weight and the right indicates MMSE scores. The abscissa indicates the comparison between NC group and three disease groups (EMCI group, LMCI group and AD group). The blue histogram represents the relief weight. The dark yellow histogram represents the MMSE values. The figure showed that the relief weights increased gradually with the reduction in MMSE scores.

It should be noted that the performance of the proposed method with EMCI and LMCI patients was relatively low (72.80 and 78.63%, respectively). These findings suggest that the hyper-network method was unable to reveal differences in network structure in the early stages of disease development. Thus, the selected network features, brain regions, or subgraph features, appear to have been insufficient for describing between-group differences effectively (the classification accuracy with single feature types was below 80%, in both the EMCI and LMCI groups). In addition, we analyzed the relief weights of features and MMSE scores among three disease groups (Figure 10). The analysis revealed that the relief weights increased gradually with the reduction in MMSE scores. This result demonstrates that the classification accuracy gradually increased with the development of the disease. The severity of the disease would be expected to enhance the differences in network structure between patients and normal controls. However, in the early stage of illness, particularly in the EMCI group, the hyper-network method was unable to reveal differences in network structure, compared with normal controls. This represents a potential limitation of the proposed method.

## Conclusion

Compared with the conventional methods of constructing functional connectivity networks, a hyper-network can reflect the interaction information among multiple brain regions and improve the classification of disease using higher-order information. However, existing methods use brain region features for classification, but an obvious deficiency of this method is that some useful topology information might be lost. To address the current limitations of conventional network modeling approaches, we proposed a method of machine learning classification combining multiple features of a hyper-network using fMRI data in AD. The proposed method has two important advantages. First, the method considers the interactions among brain regions and thus reflects more complex interactions. Second, it combines two types of complementary features for feature extraction, which ensures the integrity of the structural information and the sensitivity to change in a single brain region. The results of analyses with two different data sets showed that the proposed method improved classification performance of AD, compared with conventional methods. However, it should be noted that the proposed method was unable to identify EMCI patients because of the lack of significant structural differences of hyper-networks in these patients.

## Author contributions

HG was responsible for the study design and writing the manuscript. FZ performed data analysis and statistical processing. YX provided and integrated experimental data. JC supervised the paper. JX was the head of the funds and supervised the paper. All authors approved the final version of the manuscript.

### Conflict of interest statement

The authors declare that the research was conducted in the absence of any commercial or financial relationships that could be construed as a potential conflict of interest.
