# Supplementary material for: Machine Learning Classification Combining Multiple Features of A Hyper-Network of fMRI Data in Alzheimer's Disease
Source: Front Neurosci. 2017 Nov 21;11:615. doi: 10.3389/fnins.2017.00615 (PMC5702364; doi:10.3389/fnins.2017.00615)
Supplement: Supplementary file 1 [file Table1.PDF]

Supplemental Table T1: Results of multiple linear regression analysis between network properties and confounding variables

Confounding Variables	Coefficients	Std. Error	T Stat.	P-value	Lower 95%	Upper 95%
Clustering Coefficient HCC[1] (Adj. R_{sqr} = -0.037, P = 0.878)						
Intercept	1.308	0.046	28.412	<0.001	1.216	1.400
Gender	-0.010	0.017	-0.582	0.563	-0.045	0.025
Age	0.001	0.001	0.651	0.517	-0.001	0.002
Educational Attainments	0.000	0.007	0.052	0.958	-0.012	0.013
Clustering Coefficient HCC[2] (Adj. R_{sqr} = 0.021, P = 0.230)						
Intercept	0.838	0.106	7.929	<0.001	0.627	1.049
Gender	0.061	0.040	1.516	0.134	-0.019	0.141
Age	0.001	0.002	0.461	0.646	-0.003	0.005
Educational Attainments	0.016	0.015	1.066	0.291	-0.014	0.046
Clustering Coefficient HCC[3] (Adj. R_{sqr} = -0.003, P = 0.434)						
Intercept	0.310	0.057	5.439	<0.001	0.196	0.424
Gender	0.028	0.022	1.296	0.200	-0.015	0.071
Age	$-5.625E^{-5}$	0.001	-0.051	0.960	-0.002	0.002
Educational Attainments	0.007	0.008	0.817	0.417	-0.010	0.023

The range of age is 17–51 years. Optional values of gender are male and female. Optional values of educational attainments are illiteracy, primary school, junior high school, senior high school, junior college, college, graduate degree and above. Adj. R_{sqr}, adjusted R square. Coefficients, regression coefficient. Std. Error, standard error. T stat., T statistic. Lower 95%, low bound of 95% confidence limits. Upper 95%, upper bound of 95% confidence limits
