# Supplementary material for: Machine Learning Classification Combining Multiple Features of A Hyper-Network of fMRI Data in Alzheimer's Disease
Source: Front Neurosci. 2017 Nov 21;11:615. doi: 10.3389/fnins.2017.00615 (PMC5702364; doi:10.3389/fnins.2017.00615)
Supplement: Supplementary file 2 [file Presentation1.PDF]

Supplemental Text S1. Image Acquisition

All the subjects underwent resting state functional MRI scan using 3T MR equipment (Siemens Trio 3-Tesla scanner，Siemens, Erlangen, Germany). During the scan, subjects were instructed to relax with their eyes closed but not to fall asleep. Subjects were fitted with soft ear plugs and positioned carefully in the coil with comfortable support.

Each scan consisted of 248 contiguous EPI functional volumes (33 axial slices, repetition time (TR) = 2000 ms, echo time (TE) = 30 ms, thickness/skip = 4/0 mm, field of view (FOV) = 192×192 mm, matrix = 64×64 mm, flip angle = 90°) and the first ten volumes of time series were discarded for magnetization stabilization.
