# Supplementary material for: Machine Learning Classification Combining Multiple Features of A Hyper-Network of fMRI Data in Alzheimer's Disease
Source: Front Neurosci. 2017 Nov 21;11:615. doi: 10.3389/fnins.2017.00615 (PMC5702364; doi:10.3389/fnins.2017.00615)
Supplement: Supplementary file 3 [file Presentation2.PDF]

Supplemental Text S2. Discriminative Subgraph

As shown in the figure: +: positive samples. -: negative samples. A, B, … , F denotes respectively node in the graph. The subgraph A-B exists only in positive samples and does not exist in negative samples. The subgraph B-C exists in both positive samples and negative samples. The subgraph C-F exists only in negative samples and does not exist in positive samples. The subgraph A-B or subgraph C-F than the subgraph of B-C is more suitable for discriminative subgraphs to classify.

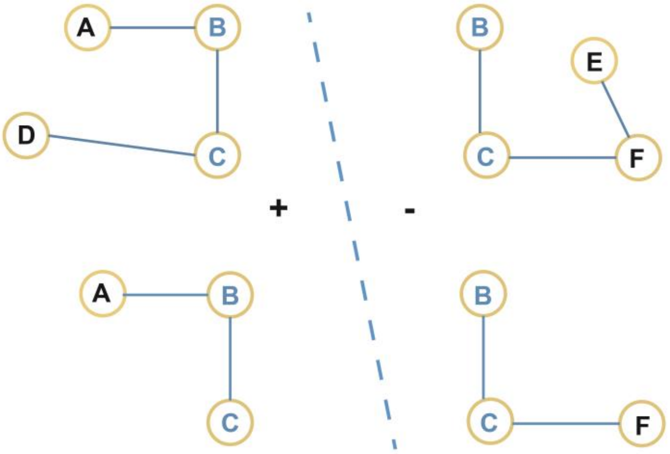

subgraph	positive	negative
A-B	√	×
B-C	√	√
C-F	×	√

The discrimination of different subgraphs
