# Supplementary material for: Machine Learning Classification Combining Multiple Features of A Hyper-Network of fMRI Data in Alzheimer's Disease
Source: Front Neurosci. 2017 Nov 21;11:615. doi: 10.3389/fnins.2017.00615 (PMC5702364; doi:10.3389/fnins.2017.00615)
Supplement: Supplementary file 4 [file Presentation3.PDF]

**Supplemental Text S3: Weisfeiler-Lehman Subtree Kernel**

Given two graphs $G$ and $H$, let $\sum_0$ be the original set of node labels of $G$ and $H$, and $\sum_i$ be the set of letters that occur as node labels at least once in $G$ or $H$ at the end of the $i-th$ iteration of the Weisfeiler-Lehman algorithm. Assume that all $\sum_i = \{\sigma_{i1}, \sigma_{i2}, \cdots, \sigma_{i|\sum_i|}\}$ are pairwise disjointed. The Weisfeiler-Lehman subtree kernel on two graphs $G$ and $H$ with $h$ iterations is defined as follows

$$k^h(G,H) = \langle \phi^h(G), \phi^h(H) \rangle$$

where

$$\phi^h(G) = \left(C_0(G,\sigma_{01}), \cdots, C_0\left(G,\sigma_{0|\sum_0|}\right), \cdots, C_h(G,\sigma_{h1}), \cdots, C_h\left(G,\sigma_{h|\sum_k|}\right)\right)$$

and

$$\phi^h(H) = \left(C_0(H,\sigma_{01}), \cdots, C_0\left(H,\sigma_{0|\sum_0|}\right), \cdots, C_h(H,\sigma_{h1}), \cdots, C_h\left(H,\sigma_{h|\sum_k|}\right)\right)$$

In this study, $C_i(G,\sigma_{ij})$ and $C_i(H,\sigma_{ij})$ are the number of occurrences of the node label $\sigma_{ij}$ in $G$ and $H$ with the $i-th$ iteration, respectively. It is noteworthy that the graph used in this study is the undirected graph.

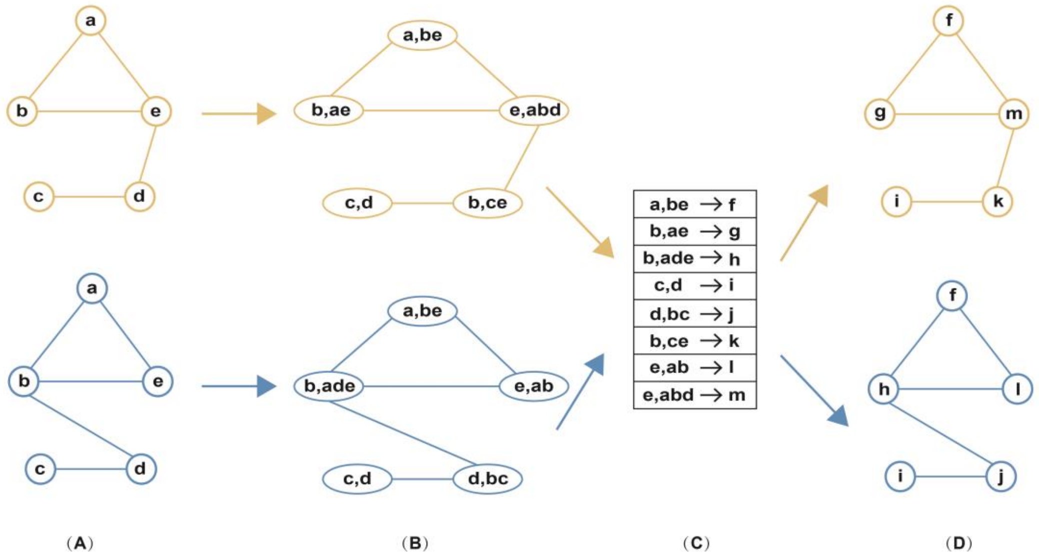


Illustration of the construction process of the WL subtree kernel

**Illustration of the construction process of the WL subtree kernel.** For given two graphs $G$ and $H$, A) the initial labeled graphs, B) augmented labels, C) label compression, D) relabeled networks. If the iteration time is set as 1, the label set is $L = \{a,b,c,d,e,f,g,h,i,j,k,l,m\}$. The $\emptyset(G) = \{1,1,1,1,1,1,1,0,1,0,1,0,1\}$and $\emptyset(H) = \{1,1,1,1,1,1,0,1,1,1,0,1,0\}$.The $k(G,H) = \langle \emptyset(G), \emptyset(H) \rangle$=7. Given two graphs, the basic process of the Weisfeiler-Lehman test is as follows: if those two graphs are unlabeled (i.e., vertices of the graph have not been assigned labels), first label each vertex with the number of edges that are connected to that vertex. Then, at each iteration step, the label of each vertex is updated based on its previous label and the labels of its neighbors. That is, compress the sorted set of updated node labels of each vertex into a new and shorter label. This process iterates until the node label sets are identical, or the number of iteration reaches its

predefined maximum value.The 1-dimensional Weisfeiler-Lehman test proceeds in iterations, which shown in Algorithm 1.

**Algorithm 1**

One iteration of the 1-dim. Weisfeiler-Lehman test of graph isomorphism

Step 1:Multiset-label determination

For i=0,set $M_i(v) = l_0(v) = l(v)$

For i>0,Assign a Multiset-label $M_i(v)$ to each node v in G and G' which consists of the Multiset $\{l_{i-1}(u)|u \in N(v)\}$

Step 2:Sorting each multiset

Sort elements in $M_i(v)$ in ascending order and concatenate them into a string $s_i(v)$

Add $l_{i-1}(v)$ as a prefix to $s_i(v)$ and call the resulting string $s_i(v)$

Step 3:Label compression

Sort all of the strings $s_i(v)$ for all v from G and G' in ascending order

Use a function $\mathrm{f}: \Sigma^* = \Sigma$ Map each string $s_i(v)$ to a new compressed label.

Step 4:Relabeling

Set $l_i(v) \coloneqq f(s_i(v))$ for all nodes in G and G'

## References


1. Shervashidze, N., et al., *Weisfeiler-Lehman Graph Kernels.* Journal of Machine Learning Research, 2011. **12**(3): p. 2539-2561.
