# Supplementary material for: Machine Learning Classification Combining Multiple Features of A Hyper-Network of fMRI Data in Alzheimer's Disease
Source: Front Neurosci. 2017 Nov 21;11:615. doi: 10.3389/fnins.2017.00615 (PMC5702364; doi:10.3389/fnins.2017.00615)
Supplement: Supplementary file 5 [file Image1.PDF]

Supplemental Figure S1. The results of subgraph features in the new data set

In the experiment, we respectively combined the subgraph features between the NC group and EMCI group. And then the same analysis were separately tested on the LMCI and AD dataset. In this figure, A denotes the differences of subgraph features between NC and EMCI, where red nodes represented the abnormal brain regions that appeared together in both groups. Therefore, these abormal brain regions between NC and EMCI were found that included right SFGdor, left MFG, right MFG, right OLF, left SFGmed, right SFGmed, left INS, left PHG, right CAL, left FFG. Similarly, B denotes the differences of subgraph features between NC and LMCI. These abnormal brain regions between them were mainly distributed in right PreCG, right SFGdor, left MFG, right MFG, left SFGmed, right SFGmed, left INS, right HIP, left PHG, right PHG, left SOG, right SOG, left MOG. C denotes the differences of subgraph features between NC and AD. These abnormal brain regions between them included left PCG, right HIP, right CUN, left LING, right LING, left FFG, right FFG, left SMG, left ANG, right ANG, left PCUN, left THA, right STG, right MTG..

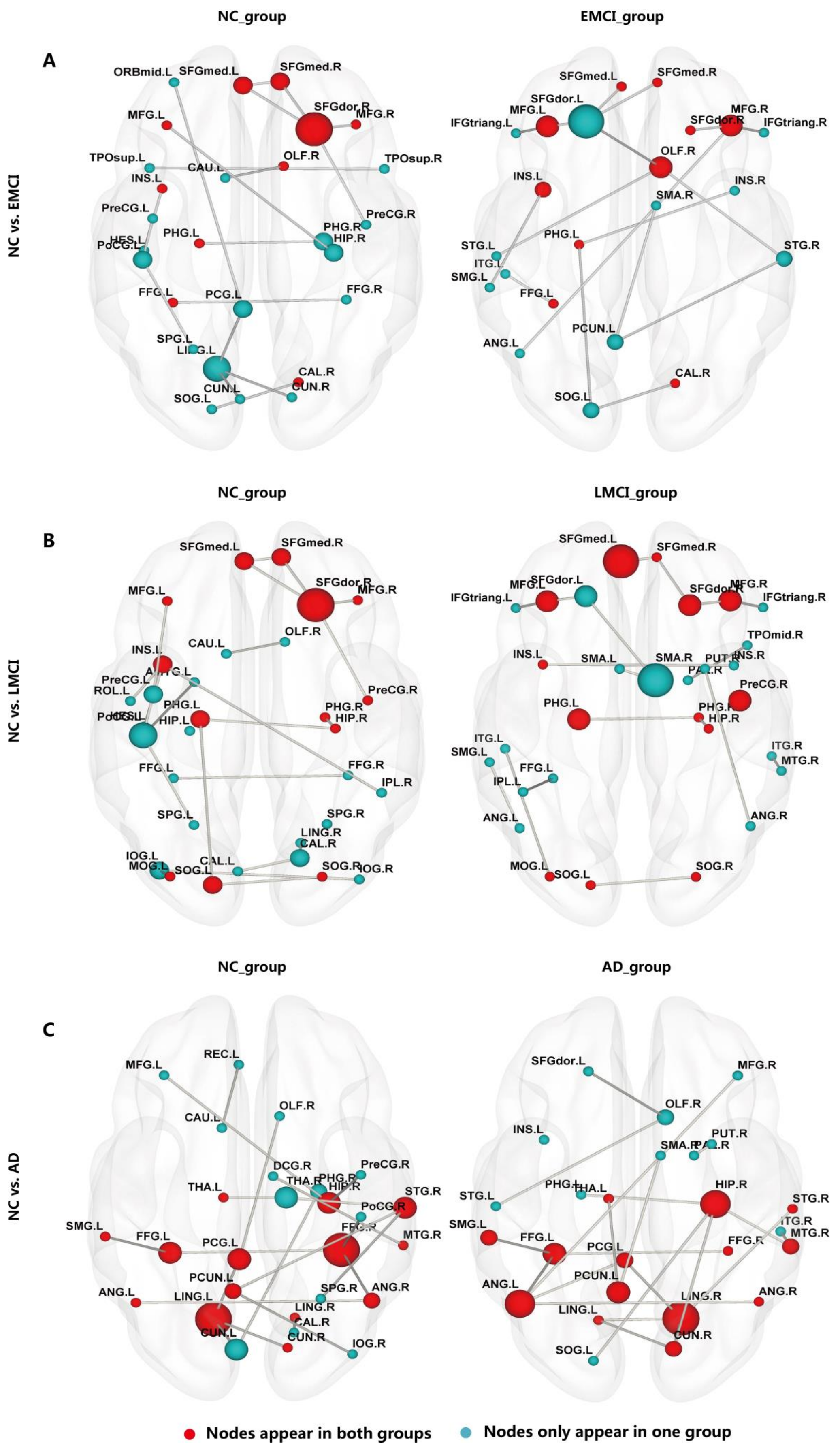

NC_group
EMCI_group
A
NC vs. EMCI
ORBmid.L
SFGmed.L
SFGmed.R
SFGdor.R
MFG.L
MFG.R
TPOsup.L
CAU.L
OLF.R
TPOsup.R
INS.L
PreCG.L
PreCG.R
HES.L
PoCG.L
PHG.L
PHG.R
HIP.R
FFG.L
PCG.L
FFG.R
SPG.L
LING.L
CAL.R
CUN.L
CUN.R
SOG.L
SFGmed.L
SFGmed.R
SFGdor.L
MFG.L
MFG.R
IFGtriang.L
SFGdor.R
IFGtriang.R
OLF.R
INS.L
INS.R
SMA.R
PHG.L
STG.L
STG.R
ITG.L
SMG.L
FFG.L
PCUN.L
ANG.L
CAL.R
SOG.L
NC_group
LMCI_group
B
NC vs. LMCI
SFGmed.L
SFGmed.R
SFGdor.R
MFG.R
MFG.L
CAU.L
OLF.R
INS.L
PreCG.L
ROL.L
PreCG.R
PHG.L
PHG.R
HIP.L
HIP.R
FFG.L
FFG.R
IPL.R
SPG.L
SPG.R
LING.R
CAL.R
IOG.L
MOG.L
CAL.L
SOG.L
SOG.R
IOG.R
SFGmed.L
SFGmed.R
MFG.L
SFGdor.L
SFGdor.R
MFG.R
IFGtriang.L
IFGtriang.R
TPOmid.R
INS.L
SMA.L
SMA.R
PUT.R
INS.R
PAL.R
PreCG.R
PHG.L
HIP.R
ITG.L
SMG.L
FFG.L
IPL.L
ITG.R
MTG.R
ANG.L
ANG.R
MOG.L
SOG.L
SOG.R
NC_group
AD_group
C
NC vs. AD
MFG.L
REC.L
CAU.L
OLF.R
DCG.R
PreCG.R
THA.L
THA.R
PHG.R
HIP.R
STG.R
PoCG.R
SMG.L
FFG.L
PCG.L
FFG.R
MTG.R
PCUN.L
SPG.R
ANG.L
LING.L
ANG.R
LING.R
CAL.R
CUN.L
CUN.R
IOG.R
SFGdor.L
MFG.R
OLF.R
INS.L
PUT.R
SMA.R
PAL.R
STG.L
PHG.L
THA.L
HIP.R
STG.R
ITG.R
SMG.L
FFG.L
PCG.L
FFG.R
MTG.R
PCUN.L
ANG.L
ANG.R
LING.R
LING.L
CUN.R
SOG.L
Nodes appear in both groups
Nodes only appear in one group
